# Tramadol’s Inhibitory Effects on Sexual Behavior: Pharmacological Studies in Serotonin Transporter Knockout Rats

**DOI:** 10.3389/fphar.2018.00676

**Published:** 2018-06-27

**Authors:** Diana C. Esquivel-Franco, Berend Olivier, Marcel D. Waldinger, Gabriel Gutiérrez-Ospina, Jocelien D. A. Olivier

**Affiliations:** ^1^Neurobiology, Groningen Institute for Evolutionary Life Sciences, University of Groningen, Groningen, Netherlands; ^2^Programa de Doctorado en Ciencias Biomédicas, Universidad Nacional Autónoma de México, Mexico City, Mexico; ^3^Departamento de Biología Celular y Fisiología, Instituto de Investigaciones Biomédicas, Universidad Nacional Autónoma de México, Mexico City, Mexico; ^4^Department of Psychopharmacology, Utrecht Institute for Pharmaceutical Sciences, Science Faculty, Utrecht University, Utrecht, Netherlands; ^5^Department of Psychiatry, Yale University School of Medicine, New Haven, CT, United States; ^6^Department of Pharmacology & Physiology, College of Medicine, Drexel University, Philadelphia, PA, United States; ^7^Coordinación de Psicobiología y Neurociencias, Facultad de Psicología, Universidad Nacional Autónoma de México, Mexico City, Mexico

**Keywords:** SSRI, sexual behavior, tramadol, rat, 5-HT_1A_ receptor, μ-opioid receptor, serotonin transporter

## Abstract

Tramadol is an effective pharmacological intervention in human premature ejaculation (PE). To investigate whether the inhibitory action of tramadol is primarily caused by its selective serotonin reuptake inhibitory (SSRI) effects we tested the dose–response effects of tramadol on sexual behavior in serotonin transporter wild type (SERT^+/+^), heterozygous (SERT^+/-^), and knockout (SERT^-/-^) rats. To investigate whether other mechanisms contribute to the inhibitory effects, WAY100,635, a 5-HT_1A_ receptor antagonist and naloxone, a μ-opioid receptor antagonist, were tested on sexual behavior together with tramadol. Tramadol dose-dependently decreases sexual activity in all genotypes. In all studies, SERT^+/-^ rats did not respond differently from SERT^+/+^ rats. WAY100,635 did not affect sexual activity in SERT^+/+^, but dose-dependently reduced sexual activity in SERT^-/-^ rats. WAY100,635 (0.3 mg/kg) combined with tramadol (20 mg/kg) significantly reduced sexual activity in SERT^+/+^ and even stronger in SERT^-/-^ rats. Naloxone did not affect sexual behavior consistently in SERT^+/+^ rats, while in SERT^-/-^ rats all doses reduced ejaculation frequency mildly. Combining naloxone (20 mg/kg) and tramadol (20 mg/kg) decreased ejaculation frequencies in both genotypes. Interestingly, combining tramadol (20 mg/kg), WAY100,635 (0.3 mg/kg) and naloxone (20 mg/kg) led to complete elimination of all sexual activity in both SERT^+/+^ and SERT^-/-^ rats. These findings suggest that the inhibitory effects of tramadol on male sexual behavior in SERT^+/+^ rats is mainly, if not exclusively, due to SERT inhibition, with an important role for 5-HT_1A_ receptors, although influence of other systems (e.g., noradrenergic) cannot be excluded. As SSRIs exert their sexual inhibition after chronic administration, tramadol may be therapeutically attractive as “on demand” therapy for PE.

## Introduction

Tramadol is a worldwide used painkiller that produces anti-nociception by activation of μ-opioid receptors (Hennies et al., 1988). Recent research has shown that tramadol is effective in treating premature ejaculation (PE) in humans (Bar-Or et al., 2012; Eassa and El-Shazly, 2013; Yang et al., 2013). Tramadol is a racemic mixture of two enantiomers (Frink et al., 1996). The first enantiomer [(+)-tramadol] and its metabolite [(+)-M_1_] are selective agonists of the μ-opioid receptor and have selective serotonin reuptake inhibitory (SSRI) effects as well; the second enantiomer [(-)-tramadol] and the (-)-M_1_ metabolite produce norepinephrine reuptake inhibition (Matthiesen et al., 1998). Due to its SSRI properties, tramadol has antidepressant-like effects (Rojas-Corrales et al., 1998, 2002). SSRIs are mainly used as antidepressants due to inhibition of the serotonin transporter (SERT), leading to an increased level of 5-HT in the synaptic cleft. However, SSRI treatment has been associated with the appearance of some serious side effects, like a decrease in the ability to reach ejaculation or orgasm (Balon, 2006); thus resulting in a significant impact on an individual’s life quality that in most cases leads to non-compliance to treatment (Higgins et al., 2010).

Previously, we found (Olivier et al., 2017a) found that tramadol inhibits sexual behavior in male rats and postulated this to be mainly due to its SSRI properties, although tramadol’s μ-opioid receptor agonistic activity might contribute in a minor way to sexual behavior inhibition. Serotonergic activation of sexual activity in male rats is primarily based on activation of 5-HT_1A_ receptors based on the pro-sexual effects observed after 5-HT_1A_ receptor agonists (Snoeren et al., 2014). Acute co-administration of a 5-HT_1A_ receptor antagonist and SSRI inhibits male rat sexual behavior, indicating that the potential sexual side effects of chronic SSRI-treatment depend on the degree of 5-HT_1A_ receptor modulation (de Jong et al., 2005a). In the present research, our rationale is to further investigate the role of 5-HT_1A_ receptor activity and μ-opioid agonist activity in tramadol’s sexual behavior inhibition. To this end, we use SERT knockout rats because they create a possibility to study the influence of tramadol on the μ-opioid system (and possibly other systems) without concomitant influence of blockade of the SERT in male sexual behavior. Although it is known that 5-HT_1A_ receptors are affected in homozygous SERT knockout (SERT^-/-^) rats (Olivier B. et al., 2010; Chan et al., 2011) practically nothing is known about changes in other systems, including the opioid system.

In the present studies, based on our previous work (Olivier et al., 2017a), we first explored several doses of tramadol (5, 10, 20, 40, and 50 mg/kg IP) on sexual behavior of wild type (SERT^+/+^), heterozygous (SERT^+/-^), or SERT^-/-^ male rats, selected, and trained for average sexual activity (2–3 ejaculations per 30-min test after a 6-weeks training period). Because we knew from previous studies (Olivier et al., 2017a) that a higher tramadol dose (40 mg/kg) in wild type rats could only marginally be influenced by naloxone, we combined a slightly inhibitory dose of tramadol (20 mg/kg) in all three genotypes with naloxone, a μ-opiate receptor antagonist. In another set of studies, we combined tramadol (20 mg/kg) with a selected, sexual behavior-inactive dose of the 5-HT_1A_ receptor antagonist WAY100,635 (0.3 mg/kg). The supporting idea of this experiment was our previous finding that combining sexually inactive doses of a 5-HT_1A_ receptor antagonist with a sexually inactive dose of an SSRI after acute administration strongly inhibits sexual behavior (de Jong et al., 2005a). Lastly, WAY100,635 (0.3 mg/kg) and naloxone (20 mg/kg) were combined with tramadol (20 mg/kg) to unravel putative modulatory effects and clarify possible interactions.

## Materials and Methods

### Animals

Wistar rats were bred in our animal facility (University of Groningen, GELIFES) using SERT^+/-^ males and females, resulting in male and female SERT^+/+^, SERT^+/-^, and SERT^-/-^ rats. Male SERT^+/+^, SERT^+/-^, and SERT^-/-^ rats of at least 12-weeks old were used for sexual behavioral experiments. Female SERT^+/+^ and SERT^+/-^ offspring were used as sexual stimulus females. Rats were housed under reversed dark-light conditions (12 h light:12 h dark, lights off from 9:00 AM to 9 PM). Animals were socially housed (2–5 per cage, maximum 4 for males). Wooden gnawing blocks and nesting material were provided for cage enrichment. Rats had *ad libitum* access to food and water. All experiments were conducted in accordance with the governmental guidelines for care and use of laboratory animals (Centrale Commissie Dierproeven). The protocol was approved by the Central Commissie Dierproeven. All efforts were made to minimize the number of animals and possible suffering.

### Female Rats

The females had double tubal ligation to prevent pregnancies. To perform the surgery, females were anesthetized (Isoflurane) and given pain relief (Fynadine, 0.1 mg/100 g) before the surgery, and 24 and 48 h after surgery. Females were at least 12-weeks old when surgery was performed, and 2 weeks of recovery were given before they were made intentionally receptive with estradiol (50 μg in 0.1 ml oil, S.C., 36–48 h before the test) for the sexual behavior training tests and experiments. Females were used once in 2 weeks and not more than two times per experimental day.

### Drug Treatment and Behavioral Experiments

A crossover-randomized design was planned in order to prevent that animals receive the same drug doses or vehicle during all the experiments, which were run over a couple of months. As described previously in Olivier et al. (2017a), when pharmacological tests were performed, male rats were given a 30-min habituation time in the test boxes right after drug administration via IP injection, before the female rat was introduced. Multiple injections were given 10 min after each other. All behavior during the 30-min test was video-recorded after introduction of the female and were also live scored and the following parameters of the first ejaculation series were deduced (Chan et al., 2010): number of ejaculations (E), number of mounts (M), number of intromissions (I), latency (s) to first mount (ML), latency (s) to first intromission (IL) and latency (s) to the first ejaculation (EL). After ejaculation, the post-ejaculatory interval (PEI) was calculated, using the time from the first ejaculation and the time of the first mount/intromission (whatever occurred first) of the second ejaculation series. Intromission ratio (IR) was calculated as: IR = (*#*I*/*(*#*I + *#*M)) *^∗^* 100%. EL was calculated using the time from the first ejaculation series minus the intromission latency of the first ejaculation series. The percentages of animals performing sexual behavior during the experimental period can be found in Supplementary Figure 2.

Because it is important to have comparable pharmacodynamics and kinetics in pharmacological studies, a test of fixed duration has been chosen; 30 min (1800 s). Parameters from only the first ejaculation series, which includes the first PEI, were used to run the statistical analysis. The rationale behind this is that some treatments can decrease sexual behavior to zero (e.g., zero ejaculations) and some animals cannot be used to perform statistics. For those cases, we used artificial values of 1800 s (i.e., the maximum test duration) for some latencies (ejaculation, mount, and intromission latency), although this is undoubtedly a matter of discussion as we have mentioned before (Chan et al., 2010; Olivier et al., 2017a). In the cases in which drugs inhibited ejaculatory behavior, few or no animal achieve a second ejaculation and statistical analyses of the second ejaculatory series was not possible. If a drug blocked ejaculation and sexual performance, data values attributed to EL, ML, IL were 1800 s and the frequencies values (MF and IF) for all animals for statistical purposes. All tables and figures show the results for the first Ejaculation Series.

### Drugs

Tramadol hydrochloride (Pharmacy, UMC Groningen, Netherlands) was prepared from tablets obtained from a local pharmacy, grinded and suspended in 0.9% NaCl (saline). Naloxone hydrochloride (Abcam; Cambridge, United Kingdom) and WAY100,635 maleate (Tocris Bioscience; Bristol, United Kingdom) were dissolved in saline and each solution was freshly prepared on each testing day. All drugs were administered via intraperitoneal (IP) injection.

### Training

After 6-weekly training tests (30 min/test), male rats were considered sexually trained and classified based on ejaculation frequencies per test in: average (2–3 ejaculations/test), fast (>3 ejaculations/test), and slow (0–1 ejaculations/test) groups (Pattij et al., 2005; Olivier et al., 2006; Chan et al., 2008). Accordingly, 95 males were sexually trained and a total of 36 male rats with an average number of ejaculations were selected and used during all the experiments, which lasted 25 weeks. In all individual experiments 12 rats per genotype [SERT^+/+^ (*N* = 32), SERT^+/-^ (*N* = 32), and SERT^-/-^ (*N* = 31)] were used and animals were used only once a week to guarantee sufficient drug washout time. Rats had a habituation period of 10 min in the testing box right before the training session. At the end of the habituation period a receptive female was introduced in the box and sexual behavior was assessed for 30 min. Females that were not receptive were switched for a different female that showed receptivity. The training and testing occurred in wooden rectangular (57 cm × 82 cm × 39 cm; glass wall) testing boxes filled with regular bedding material. To stimulate sexual behavior, bedding material was not changed during the training and testing to preserve pheromones of previous rounds and to create a more competitive sexual environment. The sexual parameters scored were: total number of ejaculations of all the tests; the total number of mounts and intromissions per ejaculatory series and their respective latencies were scored for weeks 2, 3, and 6. Only males showing stable ejaculation levels (2–3 ejaculations on the last training tests) were used for the pharmacological experiments (*N* = 12 per genotype, 36 in total). Although male SERT^-/-^ rats generally display a lower sexual behavior level than SERT^+/+^ rats (Olivier B. et al., 2010; Chan et al., 2011), we selected the highest performing SERT^-/-^ animals, determined by the number of ejaculations/test, for these experiments to avoid base-line differences that complicate drug studies. All training sessions and experiments were performed under red light conditions between 10:00 AM and 17:00 PM.

### Pharmacological Experiments

#### Experimental Design

We selected 36 male rats from a pool of 95 males that were trained weekly for 6 weeks on their basal sexual behavior. These 36 rats (12 of each genotype) did not differ in their sexual level of performance. Four experiments were performed on these 36 rats, performing one test per week on each rat. Doses of drugs and vehicle tested were distributed via a crossover design. Between experiments at least 1 week of rest (non-testing) was given.

#### Experiment 1

Tramadol dose–response. Thirty-six average ejaculating male rats were selected (*N* = 12 per genotype). The three groups went on a crossover design and received vehicle (saline), 5, 10, 20, 40, and 50-mg/kg tramadol, IP (tramadol hydrochloride). Because it was physically not possible to test 36 animals in one test-day, we performed testing per week on two consecutive testing days (always the same 2 days) over 6 weeks and randomized animals and treatment over these 2 days and over the 6 weeks.

#### Experiment 2

WAY100,635 dose–response. The same 36 animals were used on a crossover design to receive vehicle (saline), 0.1, 0.3, and 1 mg/kg WAY100,635, IP. Testing was performed over 4 weeks and two consecutive days per week.

#### Experiment 3

Naloxone dose–response. The same 36 animals were used on a crossover design received vehicle (saline), 5, 10, and 20 mg/kg naloxone, IP. Testing was performed over 4 weeks and two consecutive days per week.

#### Experiment 4

Tramadol + WAY100,635 + Naloxone. All 36 rats were treated using a crossover design with vehicle + vehicle, vehicle + tramadol (20 mg/kg), tramadol (20 mg/kg) + WAY100,635 (0.3 mg/kg), tramadol (20 mg/kg) + naloxone (20 mg/kg) or tramadol (20 mg/kg) + WAY100,635 (0.3 mg/kg) + naloxone (20 mg/kg) using IP dosing. This experiment was performed over 6 weeks and two consecutive days per week.

### Statistical Analyses

For the tramadol, WAY100,635 and naloxone dose–response experiments data were normally distributed and analyzed with parametric tests, performing one-way ANOVA with repeated measures and Bonferroni *post hoc* statistical analysis (within group) and two-way ANOVA and Dunn *post hoc* test (between groups) to analyze these data. For the last experiment (Tramadol + WAY100635 + Naloxone) data were not normally distributed and non-parametric statistics were performed; Friedman repeated measures test with a rank sum tests (Tukey-HSD) *post hoc* and a Friedman two-way ANOVA with multiple comparisons. All data were analyzed using GraphPad Prism software 6.0 (Graph Pad Software, Inc., La Jolla, CA, United States). Level of significance was set at *p* < 0.05. Parametric data are expressed as mean ± SEM and non-parametric data are expressed as median (and interquartile range).

## Results

### Sexual Stability

**FIGURE 1:**
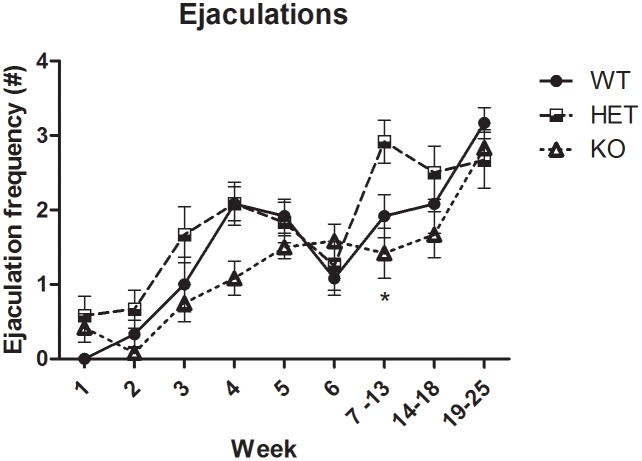
Distribution of ejaculation frequencies of male Wistar rats that were sexually trained over 6 weeks (total *n* = 95; SERT^+/+^ *n* = 32, SERT^+/-^ *n* = 32, and SERT^-/-^ *n* = 31) and pharmacologically tested from week 7 to 25 (total *n* = 36; SERT^+/+^ *n* = 12, SERT^+/-^ *n* = 12, and SERT^-/-^ *n* = 12).

The sexual performance of the animals during the duration of the study stabilized throughout the training session period. From the 95 male rats sexually trained, only 36 animals that showed sexual performance and ejaculations were selected to do the pharmacological studies (SERT^+/+^ and SERT^+/-^: 2–3 ejaculations; SERT^-/-^: 1–2 ejaculations). During this period, the number of ejaculations was registered, and animals showed stable ejaculatory behavior (**Figure 1**). The ejaculation frequencies of SERT^+/-^ animals were never significantly different from the SERT^+/+^ rats; SERT^-/-^ rats did not significantly differ in ejaculation frequencies compared to wild type rats over the whole duration of the experiments. Because SERT^+/-^ animals did not show any significant differences from SERT^+/+^ rats at any time point in any drug experiment, all results of the SERT^+/-^ animals have not been used, but all data can be found in the Supplementary Figures 1–4 and Supplementary Tables 11–14.

Because we tested the experimental animals over an extensive period of time (more than half a year) a risk of changes in the sexual performance level over time might be present. Moreover, animals received sequentially a considerable number and doses of different drugs. Although at least always 1-week wash-out was applied, the possibility exists that “carry-over” effects might occur. We did not find any evidence for either changes in the basal level of sexual behavior over time, or any “carry-over” drug effects. **Figure 1** illustrates that the “vehicle” (placebo) values (e.g., of ejaculation frequency) of the three genotype groups are very constant over time (at least 26 weeks; Supplementary Table 1). These findings are in line with our earlier experiments using tramadol (Olivier et al., 2017a) and others (Olivier et al., 2017b).

### Dose–Response of Tramadol

**FIGURE 2:**
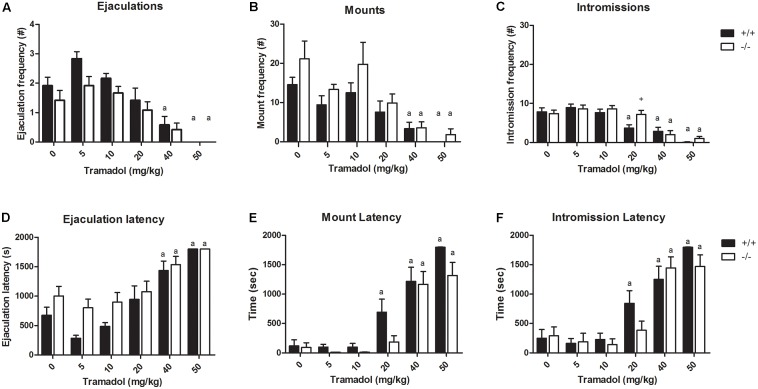
Sexual behavior of male rats (*N* = 12/group) treated with 0, 10, 20, 40, or 50 mg/kg tramadol. Data are given as mean ± SEM. The number and latency of ejaculations per 30 min **(A,D)**, number and latency of Mounts **(B,E)**, number and latency of Intromissions **(C,F)** of the first Ejaculation Series are given. Detailed statistical analyses (ANOVA repeated measures) are shown in Supplementary Tables 1, 2. a: Significant difference (*p* < 0.05) compared to saline group. +: Significant difference between SERT^+/+^ and SERT^-/-^ (*p* < 0.05).

In the dose–response experiment (Experiment 1; **Figure 2** and Supplementary Tables 3, 4) the lowest doses of tramadol (5 and 10 mg/kg) had no significant effects on sexual behavior; the intermediate and high doses (20, 40, and 50 mg/kg) induced significant effects on behavioral parameters of sexual behavior (**Figure 2**) in SERT^+/+^ animals, whereas only doses of 40 and 50 mg/kg induced significant effects in SERT^-/-^ rats. At these high doses, numbers of E, M, and I were significantly decreased and latencies of E, M, and I were significantly increased compared to the placebo (saline) group. The 50-mg/kg dose provoked a dramatic reduction of sexual activity in both SERT^+/+^ and SERT^-/-^ animals. The 20 mg/kg dose significantly increased mount and intromission latencies and significantly decreased the intromission frequency in SERT^+/+^ animals, and the number of intromissions were significantly different between genotypes.

### Dose–Response of WAY100,635

**FIGURE 3:**
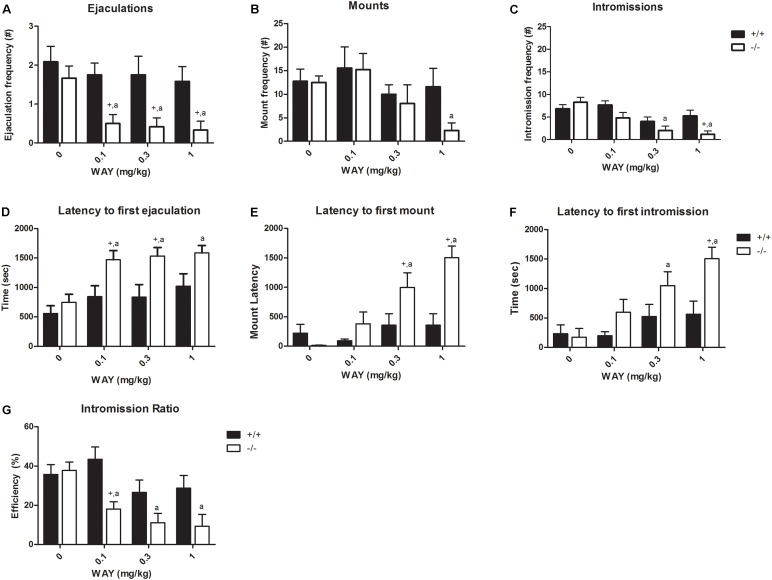
Sexual behavior of male rats (*N* = 12/group) treated with saline, WAY100,635 0.1 mg/kg, WAY100,635 0.3 mg/kg, or WAY100,635 1 mg/kg. Data are given as mean ± SEM. The number and latency of ejaculations per 30 min **(A,D)**, number and latency of Mounts **(B,E)**, number and latency of Intromissions **(C,F)**, and Copulatory Efficiency **(G)**. Detailed statistical analyses (ANOVA repeated measures) are shown in Supplementary Tables 3, 4. a: Significant difference (*p* < 0.05) compared to saline group. +: Significant difference between SERT^+/+^ and SERT^-/-^ (*p* < 0.05).

WAY100,635 (0.1, 0.3, and 1 mg/kg) had no significant effects on sexual parameters in SERT^+/+^ animals; however, in SERT^-/-^ rats all doses significantly reduced EF and increased EL (**Figure 3** and Supplementary Tables 5, 6) when compared to saline. M (at 1 mg/kg) and I (at 0.3 and 1 mg/kg) frequencies, and IR (at 0.1, 0.3, and 1 mg/kg) were significantly decreased, whereas M and I latencies (at 0.3 and 1.0 mg/kg) were significantly increased in SERT^-/-^ rats compared to SERT^+/+^ rats.

### Dose–Response of Naloxone

**FIGURE 4:**
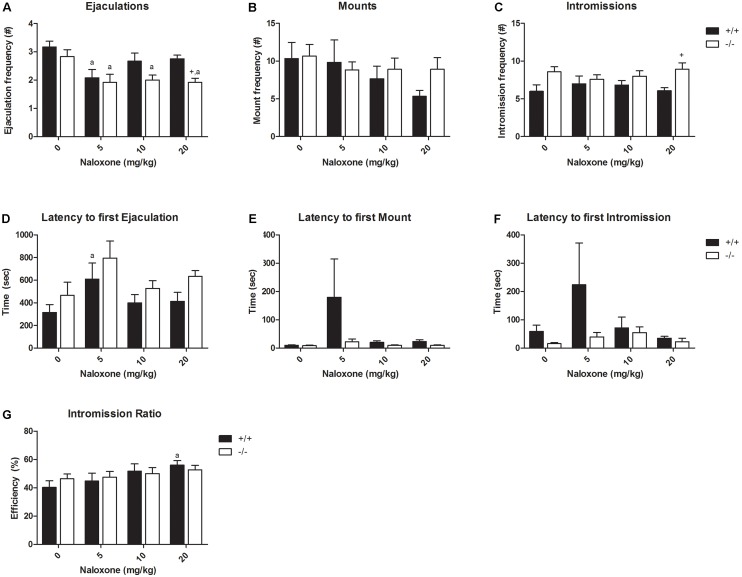
Sexual behavior of male rats (*N* = 12/group) treated with saline, Naloxone 5 mg/kg, Naloxone 10 mg/kg, or Naloxone 20 mg/kg. Data are given as mean ± SEM. The number and latency of ejaculations per 30 min **(A,D)**, number and latency of Mounts **(B,E)**, number and latency of Intromissions **(C,F)**, and Copulatory Efficiency **(G)**. Detailed statistical analyses (ANOVA repeated measures) are shown in Supplementary Tables 5, 6. a: Significant difference (*p* < 0.05) compared to saline group. +: Significant difference between SERT^+/+^ and SERT^-/-^ (*p* < 0.05).

Naloxone had very limited effects in the SERT^+/+^ animals; only the 5-mg/kg dose decreased the ejaculation frequency (**Figure 4** and Supplementary Tables 7, 8), but not in SERT^+/-^ rats. Naloxone (5, 10, and 20 mg/kg) had significant effects on the ejaculation frequency of SERT^-/-^ rats, slightly decreasing (but not in a dose-dependent way) the number of ejaculations. The rest of the parameters (M and I frequencies and latencies) was not significantly affected in either SERT^+/+^ or SERT^-/-^ animals. The efficiency to reach ejaculation (IR) was increased after 10 and 20 mg/kg naloxone administration in SERT^+/+^ animals.

### WAY100,635, Naloxone and Tramadol Interactions

**FIGURE 5:**
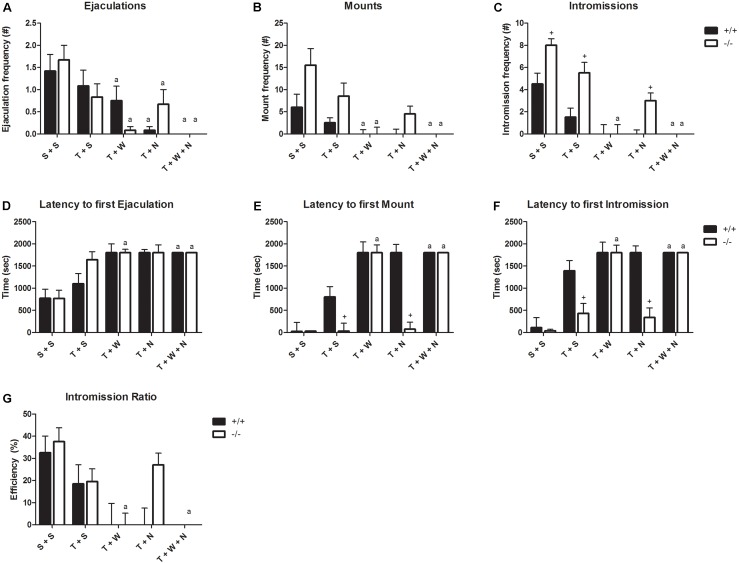
Sexual behavior of male rats (*N* = 12/group) treated with saline + saline (S + S), tramadol (20 mg/kg) + saline (T + S), tramadol (20 mg/kg) + WAY100,635 (0.3 mg/kg) (T + W), tramadol (20 mg/kg) + Naloxone (20 mg/kg; T + N), and tramadol (20 mg/kg) + WAY100,635 (0.3 mg/kg) + Naloxone (20 mg/kg; T + W + N). The number and latency of ejaculations per 30 min **(A,D)**, number and latency of Mounts **(B,E)**, number and latency of Intromissions **(C,F)**, and Intromission Rate **(G)**. Detailed statistical analyses (Friedman test) are shown in upplementary Tables 7, 8. a: Significant difference (*p* < 0.05) compared to saline + saline group. +: Significant difference between SERT^+/+^ and SERT^-/-^ groups (*p* < 0.05).

Tramadol (20 mg/kg) did not induce significant differences compared to placebo (S + S) in either SERT^+/+^ or SERT^-/-^ animals (**Figure 5** and Supplementary Tables 9, 10). Combining tramadol (20 mg/kg) with 0.3 mg/kg WAY100,635 compared to vehicle (S + S), induced significant decreases in EF and MF, and an increase in ML in SERT^+/+^ rats, whereas in SERT^-/-^ rats all parameters were significantly affected; decreases in EF, MF, IF, and IR, increases in EL, ML, and IL. However, no significant differences were present in any parameter between SERT^+/+^ and SERT^-/-^ rats. Combining tramadol (20 mg/kg) with 20 mg/kg naloxone, compared to vehicle, induced significant decreases in EF, MF, and IF and significant increases in ML and IL in SERT^+/+^ rats, whereas in SERT^-/-^ rats EF and IF were significantly decreased. Effects on IF, ML, and IL were significantly stronger in SERT^+/+^ animals compared to SERT^-/-^ animals. The combination of 0.3 mg/kg WAY100,635 + 20 mg/kg naloxone + 20 mg/kg tramadol completely abolished sexual behavior in SERT^+/+^ and SERT^-/-^ rats (**Figure 5**).

## Discussion

When administered acutely (IP), low doses of tramadol (5 and 10 mg/kg) have no significant effects on sexual behavior of SERT^+/+^, SERT^+/-^, and SERT^-/-^ male rats, selected for normal levels of ejaculation (around 2–3 ejaculations/test). The 20-mg/kg tramadol dose was clearly at the boundary of pharmacological relevance. Some minor, but inconsistent effects were seen. At doses of 40 and 50 mg/kg tramadol significantly inhibited sexual performance of all SERT genotypes, with the strongest effect at the 50-mg/kg dose that nearly reduced it to zero. These findings essentially replicated our earlier findings on the effects of tramadol on male sexual behavior in rats (Olivier et al., 2017a). We tried to gain further understanding whether the effects of tramadol on sexual behavior are consequence of the 5-HT reuptake inhibiting effects of tramadol and/or action on the μ opiate receptor. In previous investigations of our research group, performed only on wild type Wistar rats (Olivier et al., 2017a), it was hypothesized that the inhibitory effects on sexual behavior after acutely administered tramadol, were mainly due to its SSRI properties but could not exclude a role for the μ-opioid receptor (Olivier et al., 2017a). We decided to test tramadol also in SERT-knockout rats (both heterozygous and homozygous knockout animals) in order to exclude the SSRI component in the mechanism of action of tramadol in its sexual inhibiting effects. Because the effects of all drugs and doses used (tramadol, WAY100,635, naloxone and combinations) in heterozygous SERT^+/-^ rats did not essentially deviate from those in SERT^+/+^, we did not use the SERT^+/-^ data, but all information is given in Supplementary Information.

Surprisingly, tramadol exerted comparable inhibitory actions on sexual behavior in SERT^+/+^ and SERT^-/-^ rats (**Figure 2**). We had expected that tramadol would exert less inhibitory effects on sexual behavior in the SERT^-/-^ rats because of the absence of any SERT molecule in this genotype (Homberg et al., 2007). Apparently, the other main mechanism of action present in tramadol, agonism for the μ-opiate receptor, might have caused the inhibitory sexual action in the knockout rats. If so, it was expected that naloxone, a μ-opiate receptor antagonist, would antagonize the tramadol-induced decrease in sexual behavior in SERT^-/-^ rats. However, the dose of 20 mg/kg naloxone, that had limited (only inhibition of ejaculation frequency) effects in SERT^-/-^ rats on its own (Experiment 3; **Figure 4**), was neither able to antagonize the effect of tramadol on ejaculation frequency nor had any further inhibiting effects on other sexual parameters. Actually, this dose of naloxone combined with tramadol (20 mg/kg), did not change the behavior consistently compared to naloxone alone. This suggests that the sensitivity of the μ-opioid system in the brain of SERT^-/-^ rats had not been changed due to lifelong absence of the SERT. No data has been published in SERT^-/-^ rats on sensitivity of the opioid system, but in SERT^-/-^ mice the limited evidence suggests that the pharmacological sensitivity for the analgesic effects of morphine is unaltered (Hall et al., 2011). Remarkably, naloxone (**Figure 3**) had, at all doses tested in the dose–response study (5–20 mg/kg), but also in the single dose study (**Figure 5**), a very consistent, but not dose-dependent effect on ejaculation frequency in the SERT^-/-^ rats but not so in the SERT^+/+^. In our previous tramadol studies in Wild type Wistar rats (Olivier et al., 2017a), 5 and 10 mg/kg naloxone had no effect on male rat sexual behavior, whereas 20-mg/kg naloxone was more inhibitory on sexual behavior than in the present experiments. Although in the previous study (Olivier et al., 2017a) some evidence was present that a relatively low dose of naloxone (10 mg/kg) very marginally antagonized some parameters of sexual behavior (ML and EL) after a high tramadol dose (50-mg/kg) that reduced all sexual behavioral parameters, we did not repeat this experiment in the present studies because the emerging data did not point to a strong influence of the opioid system in tramadol’s effects on sexual behavior. Other ligands with μ-opiate receptor agonistic activity like morphine that inhibited male sexual behavior in rats (McIntosh et al., 1980; Ågmo and Paredes, 1988) could be, dose-dependently and completely, antagonized by naloxone. Moreover, like in our hands, the intrinsic activity of naloxone on male rat sexual behavior is not clear-cut (Gessa et al., 1979; Myers and Baum, 1979; McIntosh et al., 1980), reporting both stimulatory and inhibitory effects. Apparently, other factors contributing to the inhibitory effects of tramadol on male sexual activities are probably more important than the contribution of the opioid system.

As suggested in our previous study (Olivier et al., 2017a), we think that the inhibitory effects of tramadol on male sexual behavior in the SERT^+/+^ animals are mainly (if not exclusively) due to blockade of the SERT. When in SERT^+/+^ rats an acute dose (20 mg/kg) of tramadol that has no consistent effects on sexual behavior, is combined with 0.3 mg/kg WAY100,635, a 5-HT_1A_ receptor antagonist that also does not affect sexual behavior on its own (**Figure 5**), sexual behavior is inhibited, confirming our earlier data (Olivier et al., 2017a). It is postulated that the inhibitory action of SSRIs on male sexual behavior is mediated via 5-HT_1A_ receptors (de Jong et al., 2005a; Olivier B. et al., 2010). It is found (Bloms-Funke et al., 2011) that the SSRI component in tramadol leads to enhanced levels of extracellular concentrations of 5-HT in the brain. Actually, the (+)-enantiomer and its (+)-M_1_ metabolite of tramadol have serotonin-reuptake inhibiting effects, whereas the (-)-enantiomer and its (-)-M_2_ metabolite have noradrenalin reuptake inhibiting effects (Matthiesen et al., 1998). The latter enantiomer causes the enhanced extracellular noradrenaline levels due to blockade of the noradrenalin transporters (NET) (Bloms-Funke et al., 2011). SSRIs hardly enhance 5-HT after acute administration, e.g., in the frontal cortex (Beyer et al., 2002; Beyer and Cremers, 2008). This phenomenon possibly causes the lack of sexual inhibitory effects of SSRIs after acute administration (Olivier et al., 2017b). Pharmacological experiments with the (+)- and (-)-enantiomers of tramadol have not been performed but might be helpful in further determining the role of the noradrenergic vs. the serotonergic uptake properties of tramadol on sexual behavior. Adding a 5-HT_1A_ receptor antagonist to an SSRI leads to acute enhanced 5-HT levels in the brain (Beyer et al., 2002). These enhanced 5-HT levels very likely lead to acute and dose-dependent inhibition of male sexual behavior by SSRIs (de Jong et al., 2005a,b,c; Olivier B. et al., 2010; Olivier et al., 2017a). Our findings with tramadol are in line with the SSRI data. Lower doses of tramadol have relatively small enhancing effects on extracellular 5-HT levels in the ventral hippocampus, but higher doses have considerable enhancing effects (Bloms-Funke et al., 2011), associated with sexual inhibiting effects at high tramadol doses in our present study. The 20 mg/kg dose of tramadol has no or only very limited sexual inhibitory effects but combined with 0.3 mg/kg WAY100,635, strong inhibitory effects are found. These data strongly suggest, in analogy to SSRIs, that chronic tramadol treatment in wild type rats will lead to inhibition of male rat sexual behavior.

As shown before (Chan et al., 2011) and replicated in our studies here, SERT^-/-^ rats, but not SERT^+/-^ rats, have a basal lower level of sexual behavior. Because we wanted to study drugs on animals with comparable basal levels of sexual performance, we selected the better performing SERT^-/-^ males (*N* = 12) from the training pool (*N* = 31) after 6 weekly training sessions of 30 min (**Figure 1**). In all the pharmacology experiments (**Figures 2**–**5**) the vehicle data of these SERT^-/-^ animals never deviated significantly from the SERT^+/+^ males, indicating the stability of the two genotypes over time. Remarkably, the SERT^+/-^ rats never deviated in any aspect or under any pharmacological treatment from SERT^+/+^ rats, confirming earlier data (Chan et al., 2011). The basal level of sexual behavior in SERT^-/-^ rats is comparable to that of chronically SSRI-treated rats (de Jong et al., 2005a,b, 2006; Olivier et al., 2017b). Acute administration of SSRIs at doses that reach >80% SERT occupancy, does not reliably induce inhibition of sexual behavior. Likely, adaptations (down regulation) in the SERTs occur after chronic SSRI treatment that underlies the changed sexual performance (Oosting et al., 2016).

SERT^-/-^ rats display severe disturbances in serotonergic signaling and homeostasis (Homberg et al., 2007), including strongly reduced 5-HT tissue levels and depolarization-induced 5-HT release. Basal extracellular 5-HT levels were nine-fold increased, whereas no major adaptations in other monoaminergic systems (noradrenalin and dopaminergic systems) were found. Constitutive absence of the SERT leads to several adaptations in the serotonergic system, where in particular adaptations of 5-HT_1A_ receptors are present (Homberg et al., 2008; Olivier et al., 2008). Earlier studies in SERT^-/-^ rats with regard to male sexual behavior (Chan et al., 2011) suggested that lifelong absence of the SERT might have differential effects on two different pools of 5-HT_1A_ receptors. One pool of 5-HT_1A_ receptors with unchanged sensitivity seems to mediate the pro-sexual effects of 5-HT_1A_ receptor agonists like 8-OH-DPAT (Chan et al., 2011), whereas the other pool, mediating the inhibitory effects of blocked 5-HT_1A_ receptors, appears sensitized in the SERT^-/-^ rats (Chan et al., 2011). This complex of two differentially regulated 5-HT_1A_ receptor pools in SERT^-/-^ rats has also been found in autonomic regulation of body temperature and stress responses (Olivier et al., 2008). This pool of sensitized 5-HT_1A_ receptors leads to an enhanced response to WAY100,635, which was confirmed in the present study. WAY100,635 had no behavioral effects in SERT^+/+^ rats, but strongly (and somewhat dose-dependently) inhibited male sexual behavior at all doses in SERT^-/-^ rats. Combination of tramadol and WAY100,635 at doses that by themselves had no sexual behavior effects in SERT^+/+^ rats, led to inhibition of sexual behavior in both SERT^+/+^ and SERT^-/-^ animals, although stronger (but not significantly) in the SERT^-/-^ rats (**Figure 5**). Apparently, in SERT^+/+^ rats, adding an SSRI effect (in tramadol) to blockade of 5-HT_1A_ receptors on itself leads to sexual inhibition, whereas the tramadol + WAY100,635 combination in SERT^-/-^ animals does not inhibit more than WAY100,635 alone.

Combining tramadol (20 mg/kg) + naloxone (20 mg/kg) + WAY100,635 (0.3 mg/kg) led to severe inhibition of sexual behavior in both SERT^+/+^ and SERT^-/-^ rats (**Figure 5**). The tramadol/WAY100,635 combination in SERT^+/+^ rats has already considerable inhibitory effects, adding this to a naloxone dose (20 mg/kg) that is ineffective by itself, knocks the sexual behavior almost completely out. This holds also for the SERT^-/-^ animals. It is unclear how to explain this wiping out of all active behaviors, but behaviorally non-specific factors (sedation, motor disturbances, or otherwise) might be involved. Whether pharmacokinetic interactions might be involved is unclear, but tramadol (racemic) is extensively metabolized within the experimental period (30–60 min after injection) and rapidly, active metabolites (+ and – enantiomers) are formed (Sheikholeslami et al., 2016). In our experiments the 20 mg/kg dose played a prominent role in our behavioral studies, and this dose (Sheikholeslami et al., 2016) led to plasma and cerebrospinal fluid (CSF) levels of (+ and – tramadol) and M_1_ and M_2_ metabolites that rapidly reached peak plasma and CSF levels; plasma levels of all components were at least at a constant high level during the first 60 min, the time that we conducted our experiments. It is, however, possible that a combination of tramadol with either naloxone and/or WAY100,635 might have led to pharmacokinetic interactions, e.g., via modulation of the CYP450-mediated phase I metabolic reactions of racemic tramadol responsible for the emergence of the behaviorally active metabolites M1 and M2. In humans, phase I metabolism runs primarily via CYP2D6 and CYP3A4 (Miotto et al., 2017). Although not extensively investigated, there seems to be a large similarity between human and rat metabolism of tramadol (Sheikholeslami et al., 2016). Unfortunately, nothing is known about the exact pharmacokinetics and metabolism of WAY100,635 whereas data on naloxone are also scarce, particularly in rats. Unfortunately, this means that we cannot conclude that the combination treatments of tramadol with WAY100,635 and naloxone are influenced via metabolizing effects due to facilitation or inhibition of certain CYP450 systems in the liver or brain.

Finally, a putative role for the noradrenalin-reuptake effects of tramadol should be considered. It might be possible that NET have (partly) taken over the role of the SERT in order to compensate for the loss of the SERT in the SERT^-/-^ rat. Although there is some minor evidence for promiscuity of the NET for 5-HT (Homberg et al., 2007), there is considerable evidence (Olivier J.D.A. et al., 2010) that SERT^-/-^ rats have adapted their catecholaminergic systems to compensate for a life-long disturbed 5-HT neurotransmission and that drugs that influence such systems might have differential effects in SERT^+/+^ and SERT^-/-^ rats. Preliminary evidence with atomoxetine, a NET-inhibitor, did not point to differences in sexual behavior in SERT^+/+^ and SERT^-/-^ rats (data not shown), suggesting that the effects of tramadol in both genotypes are largely, if not exclusively due to changes in the serotonergic system.

## Conclusion

All the data gathered thus far suggest that the inhibitory action of tramadol on male sexual behavior is mainly, if not exclusively due to the blockade of the SERT, a mechanism that also in SSRIs is responsible for its inhibitory action on the ejaculation latency in males with premature ejaculation.

## Author Contributions

DE-F, BO, and JO contributed with conception and design of the work; the interpretation of the data and results, made sure all parts of the work were appropriately investigated and resolved. DE-F carried out all the experimental work, data collection and analysis, and work draft. DE-F, BO, MW, GG-O, and JO contributed on revising critically the intellectual content, accountability and accuracy of the work; provided approval for the publication of the content.

## Conflict of Interest Statement

The authors declare that the research was conducted in the absence of any commercial or financial relationships that could be construed as a potential conflict of interest.

## References

[B1] Ågmo A. Paredes R. (1988). Opioids and sexual behavior in the male rat. *Pharmacol. Biochem. Behav.* 30 1021–1034. 10.1016/0091-3057(88)90135-9 3227027

[B2] Balon R. (2006). SSRI-associated sexual dysfunction. *Am. J. Psychiatry* 163 1504–1509. 10.1176/ajp.2006.163.9.1504 16946173

[B3] Bar-Or D. Salottolo K. M. Orlando A. Winkler J. V. (2012). A randomized double-blind, placebo-controlled multicenter study to evaluate the efficacy and safety of two doses of the tramadol orally disintegrating tablet for the treatment of premature ejaculation within less than 2 minutes. *Eur. Urol.* 61 736–743. 10.1016/j.eururo.2011.08.039 21889833

[B4] Beyer C. E. Boikess S. Luo B. Dawson L. A. (2002). Comparison of the effects of antidepressants on norepinephrine and serotonin concentrations in the rat frontal cortex: an in-vivo microdialysis study. *J. Psychopharmacol.* 16 297–304. 10.1177/026988110201600403 12503828

[B5] Beyer C. E. Cremers T. I. F. H. (2008). Do selective serotonin reuptake inhibitors acutely increase frontal cortex levels of serotonin? *Eur. J. Pharmacol.* 580 350–354. 10.1016/j.ejphar.2007.11.028 18177637

[B6] Bloms-Funke P. Dremencov E. Cremers T. I. F. H. Tzschentke T. M. (2011). Tramadol increases extracellular levels of serotonin and noradrenaline as measured by in vivo microdialysis in the ventral hippocampus of freely-moving rats. *Neurosci. Lett.* 490 191–195. 10.1016/j.neulet.2010.12.049 21195741

[B7] Chan J. S. W. Olivier B. de Jong T. R. Snoeren E. M. S. Kooijman E. van Hasselt F. N. (2008). Translational research into sexual disorders: pharmacology and genomics. *Eur. J. Pharmacol* 585 426–435. 10.1016/j.ejphar.2008.02.098 18423444

[B8] Chan J. S. W. Snoeren E. M. S. Cuppen E. Waldinger M. D. Olivier B. Oosting R. S. (2011). The serotonin transporter plays an important role in male sexual behavior: a study in serotonin transporter knockout rats. *J. Sex. Med.* 8 97–108. 10.1111/j.1743-6109.2010.01961.x 20704641

[B9] Chan J. S. W. Waldinger M. D. Olivier B. Oosting R. S. (2010). Drug-induced sexual dysfunction in rats. *Curr. Protoc. Neurosci.* 53 9.34.1–9.34.11. 10.1002/0471142301.ns0934s53 20938926

[B10] de Jong T. R. Pattij T. Veening J. G. Dederen P. J. W. C. Waldinger M. D. Cools A. R. (2005a). Citalopram combined with WAY 100635 inhibits ejaculation and ejaculation-related Fos immunoreactivity. *Eur. J. Pharmacol.* 509 49–59. 10.1016/j.ejphar.2004.12.024 15713429

[B11] de Jong T. R. Pattij T. Veening J. G. Dederen P. J. W. C. Waldinger M. D. Cools A. R. (2005b). Effects of chronic paroxetine pretreatment on (±)-8-hydroxy-2-(di-n-propyl-amino)tetralin induced c-fos expression following sexual behavior. *Neuroscience* 134 1351–1361. 10.1016/j.neuroscience.2005.05.012 16019152

[B12] de Jong T. R. Pattij T. Veening J. G. Waldinger M. D. Cools A. R. Olivier B. (2005c). Effects of chronic selective serotonin reuptake inhibitors on 8-OH-DPAT-induced facilitation of ejaculation in rats: comparison of fluvoxamine and paroxetine. *Psychopharmacology* 179 509–515. 10.1007/s00213-005-2186-6 15719219

[B13] de Jong T. R. Snaphaan L. J. A. E. Pattij T. Veening J. G. Waldinger M. D. Cools A. R. (2006). Effects of chronic treatment with fluvoxamine and paroxetine during adolescence on serotonin-related behavior in adult male rats. *Eur. Neuropsychopharmacol.* 16 39–48. 10.1016/j.euroneuro.2005.06.004 16107310

[B14] Eassa B. I. El-Shazly M. A. (2013). Safety and efficacy of tramadol hydrochloride on treatment of premature ejaculation. *Asian J. Androl.* 15 138–142. 10.1038/aja.2012.96 23103596 PMC3739134

[B15] Frink M. C. Hennies H. H. Englberger W. Haurand M. Wilffert B. (1996). Influence of tramadol on neurotransmitter systems of the rat brain. *Arzneimittelforschung* 46 1029–1036. 8955860

[B16] Gessa G. L. Paglietti E. Quarantotti B. P. (1979). Induction of copulatory behavior in sexually inactive rats by naloxone. *Science* 204 203–205. 10.2307/1747606 432642

[B17] Hall F. S. Schwarzbaum J. M. Perona M. T. G. Templin J. S. Caron M. G. Lesch K. P. (2011). A greater role for the norepinephrine transporter than the serotonin transporter in murine nociception. *Neuroscience* 175 315–327. 10.1016/j.neuroscience.2010.11.057 21129446 PMC3030457

[B18] Hennies H. H. Friderichs E. Schneider J. (1988). Receptor binding, analgesic and antitussive potency of tramadol and other selected opioids. *Arzneimittelforschung* 38 877–880. 2849950

[B19] Higgins A. Nash M. Lynch A. M. (2010). Antidepressant-associated sexual dysfunction: Impact, effects, and treatment. *Drug Healthc. Patient Saf.* 2 141–150. 10.2147/DHPS.S7634 21701626 PMC3108697

[B20] Homberg J. R. De Boer S. F. Raasø H. S. Olivier J. D. A. Verheul M. Ronken E. (2008). Adaptations in pre- and postsynaptic 5-HT1Areceptor function and cocaine supersensitivity in serotonin transporter knockout rats. *Psychopharmacology* 200 367–380. 10.1007/s00213-008-1212-x 18581099

[B21] Homberg J. R. Pattij T. Janssen M. C. W. Ronken E. De Boer S. F. Schoffelmeer A. N. M. (2007). Serotonin transporter deficiency in rats improves inhibitory control but not behavioural flexibility. *Eur. J. Neurosci.* 26 2066–2073. 10.1111/j.1460-9568.2007.05839.x 17897403

[B22] Matthiesen T. Wöhrmann T. Coogan T. P. Uragg H. (1998). The experimental toxicology of tramadol: an overview. *Toxicol. Lett.* 95 63–71. 10.1016/S0378-4274(98)00023-X 9650647

[B23] McIntosh T. K. Vallano M. L. Barfield R. J. (1980). Effects of morphine, beta-endorphin and naloxone on catecholamine levels and sexual behavior in the male rat. *Pharmacol. Biochem. Behav.* 13 435–441. 10.1016/0091-3057(80)90251-8 6252562

[B24] Miotto K. Cho A. K. Khalil M. A. Blanco K. Sasaki J. D. Rawson R. (2017). Trends in tramadol: pharmacology, metabolism, and misuse. *Anesth. Analg.* 124 44–51. 10.1213/ANE.0000000000001683 27861439

[B25] Myers B. M. Baum M. J. (1979). Facilitation by opiate antagonists of sexual performance in the male rat. *Pharmacol. Biochem. Behav.* 10 615–618. 10.1016/0091-3057(79)90242-9 461486

[B26] Olivier B. Chan J. S. W. Pattij T. de Jong T. R. Oosting R. S. Veening J. G. (2006). Psychopharmacology of male rat sexual behavior: modeling human sexual dysfunctions? *Int. J. Impot. Res.* 18 S14–S23. 10.1038/sj.ijir.3901330 15843803

[B27] Olivier B. Chan J. S. W. Snoeren E. M. Olivier J. D. A. Veening J. G. Vinkers C. H. (2010). Differences in sexual behaviour in male and female rodents: role of serotonin. *Curr. Top. Behav. Neurosci.* 8 15–36. 10.1007/7854_2010_116 21374021

[B28] Olivier J. D. A. Cools A. R. Deen P. M. T. Olivier B. Ellenbroek B. A. (2010). Blockade of dopamine, but not noradrenaline, transporters produces hyperthermia in rats that lack serotonin transporters. *Eur. J. Pharmacol* 629 7–11. 10.1016/j.ejphar.2009.11.049 20004658

[B29] Olivier J. D. A. Cools A. R. Olivier B. Homberg J. R. Cuppen E. Ellenbroek B. A. (2008). Stress-induced hyperthermia and basal body temperature are mediated by different 5-HT1A receptor populations: a study in SERT knockout rats. *Eur. J. Pharmacol.* 590 190–197. 10.1016/j.ejphar.2008.06.008 18606402

[B30] Olivier J. D. A. Esquivel Franco D. C. Oosting R. Waldinger M. Sarnyai Z. Olivier B. (2017a). Tramadol: Effects on sexual behavior in male rats are mainly caused by its 5-HT reuptake blocking effects. *Neuropharmacology* 116 50–58. 10.1016/j.neuropharm.2016.11.020 27890601

[B31] Olivier J. D. A. Esquivel-Franco D. Waldinger M. Olivier B. (2017b). “Sexual dysfunction, depression and antidepressant: a translational approach,” in *Sexual Dysfunction*, ed. Pesek K. (Rijeka: InTech), 59–76. 10.5772/711

[B32] Oosting R. S. Chan J. S. Olivier B. Banerjee P. Choi Y. K. Tarazi F. (2016). Differential effects of vilazodone versus citalopram and paroxetine on sexual behaviors and serotonin transporter and receptors in male rats. *Psychopharmacology* 233 1025–1034. 10.1007/s00213-015-4198-1 26758283 PMC4759230

[B33] Pattij T. de Jong T. R. Uitterdijk A. Waldinger M. D. Veening J. G. Cools A. R. (2005). Individual differences in male rat ejaculatory behaviour: searching for models to study ejaculation disorders. *Eur. J. Neurosci.* 22 724–734. 10.1111/j.1460-9568.2005.04252.x 16101754

[B34] Rojas-Corrales M. O. Berrocoso E. Gibert-Rahola J. Micó J. A. (2002). Antidepressant-like effects of tramadol and other central analgesics with activity on monoamines reuptake, in helpless rats. *Life Sci.* 72 143–152. 10.1016/S0024-3205(02)02220-8 12417248

[B35] Rojas-Corrales M. O. Gibert-Rahola J. Micó J. A. (1998). Tramadol induces antidepressant-type effects in mice. *Life Sci.* 63 L175–L180. 10.1016/S0024-3205(98)00369-5 9749830

[B36] Sheikholeslami B. Gholami M. Lavasani H. Rouini M. (2016). Evaluation of the route dependency of the pharmacokinetics and neuro-pharmacokinetics of tramadol and its main metabolites in rats. *Eur. J. Pharm. Sci.* 92 55–63. 10.1016/j.ejps.2016.06.021 27365222

[B37] Snoeren E. M. S. Veening J. G. Olivier B. Oosting R. S. (2014). Serotonin 1A receptors and sexual behavior in male rats: a review. *Pharmacol. Biochem. Behav.* 121 102–114. 10.1016/j.pbb.2013.11.007 24239787

[B38] Yang L. Qian S. Liu H. Liu L. Pu C. Han P. (2013). Role of tramadol in premature ejaculation: a systematic review and meta-analysis. *Urol. Int.* 91 197–205. 10.1159/000348826 23751284

