# Supplementary material for: Tramadol’s Inhibitory Effects on Sexual Behavior: Pharmacological Studies in Serotonin Transporter Knockout Rats
Source: Front Pharmacol. 2018 Jun 27;9:676. doi: 10.3389/fphar.2018.00676 (PMC6030355; doi:10.3389/fphar.2018.00676)
Supplement: Supplementary file 1 [file Table_1.PDF]

Suppl. table 1: Sexual Behavior performance over time of male SERT$^{+/+}$, SERT$^{+/-}$ and SERT$^{-/-}$ Wistar rats. N=12/group

	Week									
SERT	1<br>Mean ± SEM<br>**A**	2<br>Mean ± SEM<br>**B**	3<br>Mean ± SEM<br>**C**	4<br>Mean ± SEM<br>**D**	5<br>Mean ± SEM<br>**E**	6<br>Mean ± SEM<br>**F**	7-13<br>Mean ± SEM<br>**G**	14-18<br>Mean ± SEM<br>**H**	19-25<br>Mean ± SEM	ANOVA repeated measures
+/+	0.0±0.0	0.33±0.18	1.0±0.36<br>**A,B**	2.08±0.28<br>**A,B**	1.91±0.2	1.08± 0.22	1.91±0.28<br>**A,B**	2.08±0.3<br>**A,B**	3.16±0.2<br>**A,B,C,E, F,G**	$F_{(8.88)}$=15.23; P<0.0001
+/-	0.58±0.25	0.66±0.25	1.66±0.3	2.08±0.22<br>**A,B**	1.83±0.2	1.25±0.32	2.91±0.28<br>**A,B,F**	2.50±0.3<br>**A,B**	2.66±0.3<br>**A,B,F**	$F_{(8.88)}$= 8.94; P<0.0001
-/-	0.41±0.19	0.08±0.08	0.75±0.2	1.08±0.22	1.50±0.1<br>**A,B**	1.58±0.22<br>**A,B**	1.41±0.33<br>**B**	1.66±0.3<br>**A,B**	2.83±0.2<br>**A,B,C,D ,E,F,G, H**	$F_{(8.88)}$= 14.05; P<0.0001
2-way ANOVA	**ns**	**ns**	**ns**	**ns**	**ns**	**ns**	**P<0.001**	**ns**	**ns**	$F_{(8.297)}$= 28.05; P<0.001
