# Supplementary material for: Tramadol’s Inhibitory Effects on Sexual Behavior: Pharmacological Studies in Serotonin Transporter Knockout Rats
Source: Front Pharmacol. 2018 Jun 27;9:676. doi: 10.3389/fphar.2018.00676 (PMC6030355; doi:10.3389/fphar.2018.00676)
Supplement: Supplementary file 2 [file Table_2.PDF]

Suppl. table 2: Percentage on animals performing sexual behavior parameters during drugs administration. $SERT^{+/+}$ $SERT^{-/-}$ and $SERT^{+/-}$ Wistar rats. N=12/group

Dose of tramadol, mg/kg	Parameters	0 mg/kg	5 mg/kg	10 mg/kg	20 mg/kg	40 mg/kg	50 mg/kg
SERT		%	%	%	%	%	%
+/+<br>-/-<br>+/-	*Ejaculation*	91.6<br>75<br>100	100<br>91.6<br>91.6	100<br>100<br>100	58.3<br>66.6<br>83.3	33.3<br>25<br>33.3	0<br>0<br>25
+/+<br>-/-<br>+/-	*Mount*	100<br>100<br>100	100<br>100<br>100	100<br>100<br>100	75<br>100<br>91.6	41.6<br>41.6<br>41.6	0<br>33.3<br>8.3
+/+<br>-/-<br>+/-	*Intromission*	100<br>100<br>100	100<br>91.6<br>100	100<br>100<br>100	75<br>91.6<br>83.3	41.6<br>25<br>41.6	0<br>33.3<br>25
Dose of naloxone, mg/kg	Parameters	0 mg/kg	5 mg/kg	10 mg/kg	20 mg/kg		
SERT		%	%	%	%		
+/+<br>-/-<br>+/-	*Ejaculation*	100<br>100<br>100	91.6<br>91.6<br>91.6	100<br>100<br>100	100<br>100<br>91.6		
+/+<br>-/-<br>+/-	*Mount*	100<br>100<br>100	100<br>100<br>100	100<br>100<br>100	100<br>100<br>91.6		
+/+<br>-/-<br>+/-	*Intromission*	100<br>100<br>100	91.6<br>100<br>100	100<br>100<br>100	100<br>100<br>91.6		
Dose of WAY100-635, mg/kg	Parameters	0 mg/kg	0.1 mg/kg	0.3 mg/kg	1 mg/kg		
SERT		%	%	%	%		
+/+<br>-/-<br>+/-	*Ejaculation*	75<br>91.6<br>91.6	83.3<br>33.3<br>91.6	66.6<br>25<br>66.6	66.6<br>16.6<br>66.6		
+/+<br>-/-<br>+/-	*Mount*	91.6<br>100<br>91.6	100<br>91.6<br>100	91.6<br>41.6<br>83.3	75<br>25<br>83.3		
+/+<br>-/-<br>+/-	*Intromission*	91.6<br>91.6<br>91.6	100<br>75<br>100	75<br>41.6<br>83.3	83.3<br>25<br>83.3		
Dose of drug, mg/kg	Parameters	0 mg/kg (saline + saline)	20 mg/kg Tramadol + Saline	20 mg/kg Tramadol + WAY 100635	20 mg/kg Tramadol + Naloxone	20 mg/kg Tramadol + WAY 100635 +	

						Naloxone
SERT		%	%	%	%	%
+/+	*Ejaculation*	58.3	50	33.3	8.3	0
-/-		83.3	50	8.3	33.3	0
+/-		83.3	41.6	33.3	16.6	0
+/+	*Mount*	83.3	66.6	25	33.3	0
-/-		100	91.6	25	91.6	0
+/-		100	66.6	41.6	50	0
+/+	*Intromission*	83.3	50	41.6	16.6	0
-/-		100	66.6	16.6	75	0
+/-		100	58.3	58.3	58.3	0
