# Supplementary material for: Tramadol’s Inhibitory Effects on Sexual Behavior: Pharmacological Studies in Serotonin Transporter Knockout Rats
Source: Front Pharmacol. 2018 Jun 27;9:676. doi: 10.3389/fphar.2018.00676 (PMC6030355; doi:10.3389/fphar.2018.00676)
Supplement: Supplementary file 3 [file Table_3.PDF]

Suppl. table 3: Effects of Tramadol on Sexual Behavior of male SERT$^{+/+}$ Wistar rats.

N=12/group

Dose of tramadol, mg/kg	0 mg/kg A	5 mg/kg B	10 mg/kg C	20 mg/kg D	40 mg/kg E	50 mg/kg	ANOVA repeated measures significance
Parameters measured	Mean ± SEM	Mean ± SEM	Mean ± SEM	Mean ± SEM	Mean ± SEM	Mean ± SEM	
# E	1.92±0.28	2.83±0.24	2.16±0.16	1.42±0.41 B	0.58±0.28 A,B,C	0.0±0.0 A,B,C,D	F(5,11)=17.29; P<0.0001
Latency 1st M (s)	119.8±102.8	98.5±48.46	99.1±61.66	692.8±222.1 A	1217±238.9 A,B,C	1795±5.35 A,B,C,D	F(5,11)=24.38; P<0.0001
Latency 1st I (s)	249.9±147	161.9±84.23	229.3±106.2	843.6±214.5 A,B	1249±225.5 A,B,C	1795±5.34 A,B,C,D	F(5,11)=18.26; P<0.0001
# M 1st series	14.58±1.8	9.42±2.31	12.50±2.6	7.58±2.83 B	3.33±1.68 A,C	0.00±0.00 A,B,C	F(5,11)=8.136; P<0.0001
# I 1st series	7.83±1.06	8.92±0.93	7.67±0.87	3.66±0.88 A	2.83±1.06 A,B,C	0.08±0.08 A,B,C	F(5,11)=14.61; P<0.0001
Latency 1st E (s)	773.9±142	448.4±92.45	714.9±115.1	1107±195.3 B	1503±133.6 A,B,C	1800±0.0 A,B,D	F(5,11)=17.21; P<0.0001
PEI	425.9±22.99	394.6±23.65	440.4±21.63	399.7±56.88	493.3±53.64	-------------	--------------------
CE_1	34±4.85	54.92±6.30	43±5.22	37.92±9.6	21.83±8.41 B	8.33±8.33 B,C	F(5,11)=4.650;P= 0.0013

M= Mount; I= Intromission; E= Ejaculation; PEL= post-ejaculatory interval; #= number; CE= copulatory efficiency = [# intromissions / (# intromissions + # mounts)]*100. A= Significantly (P<0.05) different from 0 mg/kg. B= Significantly (P<0.05) different from 5 mg/kg. C= Significantly (P<0.05) different from 10 mg/kg. D= Significantly (P<0.05) different from 20 mg/kg. E= Significantly (P<0.05) different from 40 mg/kg.
