# Supplementary material for: Tramadol’s Inhibitory Effects on Sexual Behavior: Pharmacological Studies in Serotonin Transporter Knockout Rats
Source: Front Pharmacol. 2018 Jun 27;9:676. doi: 10.3389/fphar.2018.00676 (PMC6030355; doi:10.3389/fphar.2018.00676)
Supplement: Supplementary file 4 [file Table_4.PDF]

Suppl. table 4: Effects of Tramadol on Sexual Behavior of male SERT$^{-/-}$ Wistar rats.

N=12/group

Dose of tramadol, mg/kg	0 mg/kg A	5 mg/kg B	10 mg/kg C	20 mg/kg D	40 mg/kg E	50 mg/kg	ANOVA repeated measures significance
Parameters measured	Mean ± SEM	Mean ± SEM	Mean ± SEM	Mean ± SEM	Mean ± SEM	Mean ± SEM	
# E	1.42±0.33	1.92±0.31	1.66±0.22	1.08±0.29	0.41±0.22 B,C	0.0±0.0 A,B,C,D	F(5,11)=9.643; P<0.0001
Latency 1st M (s)	91.760±80.11	11.41±2.28	12.11±4.45	184.1±109.2	1165±220 A,B,C	1316±225.5 A,B,C,D	F(5,11)=17.88; P<0.0001
Latency 1st I (s)	290.8±150	186.7±147.2	141.3±98.39	386.4±152.9	1445±189.7 A,B,C,D	1469±198 A,B,C,D	F(5,11)=17.98; P<0.0001
# M 1st series	21.17±4.53	13.33±1.32	19.75±5.62	9.91±2.26	3.58±1.49 A,C	1.83±1.48 A,C	F(5,11)=6.58; P<0.0001
# I 1st series	7.41±0.84	8.58±1.04	8.58±0.89	7.16±1.07	2.0±1.05 A,B,D	1.0±0.53 A,B,D	F(5,11)=15.35; P<0.0001
Latency 1st E (s)	1062±162.6	840.6±141.6	939.5±1607	1180±163.3	1630±103.1 A,B,C	1800±0.0 A,B,C,D	F(5,11)=9.16; P<0.0001
PEI	485.6±28.25	476.3±36.58	503.2±22.26	521.5±58.44	506.1±90.66	-------------	-----------------
CE_1	31.33±4.52	37.33±4.0	40.75±5.55	43.25±6.15	11.33±5.98 C,D	20.83±10.13	F(5,11)=4.16;P=0.0028

M= Mount; I= Intromission; E= Ejaculation; PEL= post-ejaculatory interval; #= number; CE= copulatory efficiency = [# intromissions / (# intromissions + # mounts)] *100. A= Significantly (P<0.05) different from 0 mg/kg. B= Significantly (P<0.05) different from 5 mg/kg. C= Significantly (P<0.05) different from 10 mg/kg. D= Significantly (P<0.05) different from 20 mg/kg. E= Significantly (P<0.05) different from 40 mg/kg.
