# Supplementary material for: Tramadol’s Inhibitory Effects on Sexual Behavior: Pharmacological Studies in Serotonin Transporter Knockout Rats
Source: Front Pharmacol. 2018 Jun 27;9:676. doi: 10.3389/fphar.2018.00676 (PMC6030355; doi:10.3389/fphar.2018.00676)
Supplement: Supplementary file 5 [file Table_5.PDF]

Suppl. table 5: Effects of WAY10065 on Sexual Behavior of male $SERT^{+/+}$ Wistar rats.

N=12/group

Dose of WAY10065, mg/kg	0 mg/kg A	1.1. mg/kg B	1.3. mg/kg C	1 mg/kg	ANOVA repeated measures significance
Parameters measured	Mean ± SEM	Mean ± SEM	Mean ± SEM	Mean ± SEM	
# E	2.08±0.39	1.75±0.30	1.75±0.47	1.58±0.37	F(3,11)=0.4419; P=0.7246
Latency 1st M (s)	219±151.6	94.17±30.77	356.8±195	358.8±196.1	F(3,11)=1.220; P=0.3178
Latency 1st I (s)	230.3±151.4	196.9±70.15	523.7±206.6	561.2±224.2	F(3,11)=1.86; P=0.1542
# M 1st series	12.75±2.60	15.58±4.47	10.83±2.47	11.58±3.92	F(3,11)=0.331; P=0.8025
# I 1st series	6.83±0.88	7.66±0.91	4.83±1.021	5.25±1.28	F(3,11)=1.75; P=0.1746
Latency 1st E (s)	637.8±137	963.3±185.4	901.9±201.3	1056±202.9	F(3,11)=1.644; P=0.1980
PEI	335.6±40.61	438.8±45.25	359.1±34.96	378.9±15.94	F(3,11)= 1.467; P=0.2440
CE_1	35.75±4.93	43.42±6.28	26.50±6.35	28.75±6.35	F(3,11)=3.14;P=0.381

M= Mount; I= Intromission; E= Ejaculation; PEL= post-ejaculatory interval; #= number; CE= copulatory efficiency = [# intromissions / (# intromissions + # mounts)] *100. A= Significantly (P<0.05) different from 0 mg/kg. B= Significantly (P<0.05) different from 0.1 mg/kg. C= Significantly (P<0.05) different from 0.3 mg/kg.
