# Supplementary material for: Tramadol’s Inhibitory Effects on Sexual Behavior: Pharmacological Studies in Serotonin Transporter Knockout Rats
Source: Front Pharmacol. 2018 Jun 27;9:676. doi: 10.3389/fphar.2018.00676 (PMC6030355; doi:10.3389/fphar.2018.00676)
Supplement: Supplementary file 6 [file Table_6.PDF]

Suppl. table 6: Effects of WAY10065 on Sexual Behavior of male SERT$^{-/-}$ Wistar rats.

N=12/group

Dose of WAY10065, mg/kg	1. mg/kg A	0.1 mg/kg B	0.3 mg/kg C	1 mg/kg	ANOVA repeated measures significance
Parameters measured	Mean ± SEM	Mean ± SEM	Mean ± SEM	Mean ± SEM	
# E	1.667±0.30	0.500±0.23 A	0.416±0.22 A	0.333±0.22 A	F(3,11)=6.950; P=0.0009
Latency 1st M (s)	13.92±3.99	381.1±199.4	995.9±254.9 A	1502±200.9 A,B	F(3,11)=13.41; P=0.0001
Latency 1st I (s)	173.2±148.1	595.3±219.4	1049±237.1 A	1505±198.9 A,B	F(3,11)=10.68; P=0.0001
# M 1st series	12.50±1.390	15.25±3.43	8.333±4.157	2.33±1.597 A,B	F(3,11)=5.024; P=0.0056
# I 1st series	8.250±1.109	4.833±1.205	2.583±1.158 A	1.167±0.767 A,B	F(3,11)=8.971; P=0.0002
Latency 1st E (s)	769.8±136.8	1549±117.6 A	1569±123.2 A	1590±124.6 A	F(3,11)=8.277; P=0.0003
PEI	471.5±45.77	459.5±139.5	383.5±16.50	310.0±0.0	---------------------------------
CE₁	37.83±4.206	18.08±3.747 A	11.08±4.864 A	9.33±6.00 A	F(3,11)=9.26;P=0.0001

M=Mount; I= Intromission; E= Ejaculation; PEL= post-ejaculatory interval; #= number; CE= copulatory efficiency = [# intromissions / (# intromissions + # mounts)]*100. A= Significantly (P<0.05) different from 0 mg/kg. B= Significantly (P<0.05) different from 0.1 mg/kg. C= Significantly (P<0.05) different from 0.3mg/kg.
