# Supplementary material for: Tramadol’s Inhibitory Effects on Sexual Behavior: Pharmacological Studies in Serotonin Transporter Knockout Rats
Source: Front Pharmacol. 2018 Jun 27;9:676. doi: 10.3389/fphar.2018.00676 (PMC6030355; doi:10.3389/fphar.2018.00676)
Supplement: Supplementary file 7 [file Table_7.PDF]

Suppl. table 7: Effects of Naloxone on Sexual Behavior of male SERT$^{+/+}$ Wistar rats.

N=12/group

Dose of Naloxone, mg/kg	0 mg/kg A	5 mg/kg B	10 mg/kg C	20 mg/kg	ANOVA repeated measures significance
Parameters measured	Mean ± SEM	Mean ± SEM	Mean ± SEM	Mean ± SEM	
# E	3.167±0.2072	2.083±0.2876 A	2.667±0.2843	2.750±0.1306	F(3,11)=5.141; P=0.0050
Latency 1st M (s)	10.00±1.467	179.5±135.4	20.25±5.783	23.58±6.129	F(3,11)=1.461; P=0.2430
Latency 1st I (s)	58.92±22.16	223.9±147.3	71.58±38.27	35.08±6.516	F(3,11)=1.353; P=0.2741
# M 1st series	10.33±2.140	9.833±2.972	7.667±1.671	5.333±0.8009	F(3,11)=1.616; P=0.2040
# I 1st series	6.000±0.8704	7.000±1.030	6.833±0.6134	6.083±0.3981	F(3,11)=0.527; P=0.6664
Latency 1st E (s)	315.7±68.62	610.8±140.1 A	400.7±72.42	413.8±79.19	F(3,11)=3.556; P=0.0246
PEI	324.1±17.32	410.4±33.80	386.3±18.78	385.8±25.88	F(3,11)= 2.350; P=0.0860
CE_1	40.33±4.588	44.67±5.602	51.75±5.133	56.00±3.231 A	F(3,11)=5.00;P=0.0057

M= Mount; I= Intromission; E= Ejaculation; PEL= post-ejaculatory interval; #= number; CE= copulatory efficiency = [# intromissions / (# intromissions + # mounts)]*100. A= Significantly (P<0.05) different from 0 mg/kg. B= Significantly (P<0.05) different from 5 mg/kg. C= Significantly (P<0.05) different from 10 mg/kg.
