# Supplementary material for: Tramadol’s Inhibitory Effects on Sexual Behavior: Pharmacological Studies in Serotonin Transporter Knockout Rats
Source: Front Pharmacol. 2018 Jun 27;9:676. doi: 10.3389/fphar.2018.00676 (PMC6030355; doi:10.3389/fphar.2018.00676)
Supplement: Supplementary file 8 [file Table_8.PDF]

Suppl. table 8: Effects of Naloxone on Sexual Behavior of male SERT$^{-/-}$ Wistar rats.

N=12/group

Dose of Naloxone, mg/kg	0 mg/kg	5 mg/kg	10 mg/kg	20 mg/kg	ANOVA repeated measures significance
Parameters measured	Mean ± SEM	Mean ± SEM	Mean ± SEM	Mean ± SEM	
# E	2.833±0.2410	1.917±0.2876 A	2.000±0.1741 A	1.917±0.1486 A	F(3,44)=5.315; P=0.0042
Latency 1st M (s)	8.750±1.638	22.00±10.41	9.583±1.948	9.750±1.629	F(3,44)=1.320; P=0.2844
Latency 1st I (s)	15.75±4.129	39.58±15.22	54.42±20.62	22.42±12.40	F(3,44)=1.397; P=0.2611
# M 1st series	10.67±1.534	8.833±1.065	8.917±1.495	8.917±1.540	F(3,44)=0.416; P=0.742
# I 1st series	8.583±0.6681	7.583±0.6088	8.000±0.7177	8.917±0.8480	F(3,44)=0.800; P=0.502
Latency 1st E (s)	465.6±117.2	795.5±150.7	527.1±68.98	633.8±51.73	F(3,44)=2.623; P=0.0669
PEI	341.0±21.91	411.3±36.91	463.9±34.07	436.8±35.51	F(3,44)= 2.686; P=0.0594
CE_1	46.33±3.363	47.50±4.093	50.00±4.230	52.67±3.170	F(3,44)=0.5414;P=0.6573

M= Mount; I= Intromission; E= Ejaculation; PEL= post-ejaculatory interval; #= number; CE= copulatory efficiency = [# intromissions / (# intromissions + # mounts)]*100. A= Significantly (P<0.05) different from 0 mg/kg. B= Significantly (P<0.05) different from 5 mg/kg. C= Significantly (P<0.05) different from 10 mg/kg.
