# Supplementary material for: Tramadol’s Inhibitory Effects on Sexual Behavior: Pharmacological Studies in Serotonin Transporter Knockout Rats
Source: Front Pharmacol. 2018 Jun 27;9:676. doi: 10.3389/fphar.2018.00676 (PMC6030355; doi:10.3389/fphar.2018.00676)
Supplement: Supplementary file 9 [file Table_9.PDF]

Suppl. table 9: WAY,100635 + naloxone + tramadol effects in male $SERT^{+/+}$ rats. N=12 animals/group

Dose of drug, mg/kg IP	0 mg/kg (saline + saline) A	20 mg/kg Tramadol + Saline B	20 mg/kg Tramadol + WAY 100635 C	20 mg/kg Tramadol + Naloxone D	20 mg/kg Tramadol + WAY 100635 + Naloxone	Friedman test significance
Parameters	Median (IQR)	Median (IQR)	Median (IQR)	Median (IQR)	Median (IQR)	
# E	2.000(2.75)	0.5000(2)	0.0(2) A	0.0(0) A	0.0(0.0) A	F(4,11)=4.576; P=0.0035
Latency 1st M (s)	27.00(66.5)	802.0(1699)	1800(1682)	1800(869)	1800(0.0) A	F(4,11)=20.63; P=0.0004
Latency 1st I (s)	108.59(1569)	1390(1576)	1800(1679)	1800(0)	1800(0.0) A	F(4,11)=19.60; P=0.0006
# M 1st series	6.000(14.75)	2.500(5)	0.0(2.75) A	0.0(1.75)	0.0(0.0) A	F(4,11)=25.31; P<0.0001
# I 1st series	4.500(6)	1.500(5)	0.0(5)	0.0(0)	0.0(0.0) A	F(4,11)=18.14; P=0.0012
Latency 1st E (s)	771.5(1476.5)	1101(1538)	1800(1325)	1800(0)	1800(0.0) A	F(4,11)=4.598; P=0.0034
PEI	423.0(69)	423.0(157)	467.5(325)	--------------	------------	F(2,11)=2.439; P=0.2954
CE_1	32.50(56.5)	18.50(54.50)	0.0(44.75)	0.0(0)	0.0(0.0)	F(4,11)=13.86; P=0.0078

M= Mount; I= Intromission; E= Ejaculation; PEL= post-ejaculatory interval; #= number; CE= copulatory efficiency = [# intromissions / (# intromissions + # mounts)] *100

A= Significantly (P<0.05) different from 0 mg/kg. B= Significantly (P<0.05) different from saline + tramadol (20 mg/kg). C= Significantly (P<0.05) different from tramadol (20 mg/kg) + WAY100,635 (0.3 mg/kg). D= Significantly (P<0.05) different from tramadol (20 mg/kg) + naloxone (20 mg/kg).
