# Supplementary material for: Tramadol’s Inhibitory Effects on Sexual Behavior: Pharmacological Studies in Serotonin Transporter Knockout Rats
Source: Front Pharmacol. 2018 Jun 27;9:676. doi: 10.3389/fphar.2018.00676 (PMC6030355; doi:10.3389/fphar.2018.00676)
Supplement: Supplementary file 10 [file Table_10.PDF]

Suppl. table 10: WAY,100635 + naloxone + tramadol effects in male SERT$^{-/-}$ rats. N=12 animals/group

Dose of drug, mg/kg IP	0 mg/kg (saline + saline) A	20 mg/kg Tramadol + Saline B	20 mg/kg Tramadol + WAY 100635 C	20 mg/kg Tramadol + Naloxone D	20 mg/kg Tramadol + WAY 100635 + Naloxone	Friedman test significance
Parameters	Median (IQR)	Median (IQR)	Median (IQR)	Median (IQR)	Median (IQR)	
# E	1.500(1.5)	0.5000(1.75)	0.0(0) A	0.0(1) A	0.0(0.0) A	F(4,11)=8.579; P<0.0001
Latency 1st M (s)	27.50(31.5)	33.00(498)	1800(591) A,B	74.00(473)	1800(0.0) A,B,D	F(4,11)=36.46; P<0.0001
Latency 1st I (s)	35.50(66.75)	430.0(1655)	1800(0) A	339.0(1488)	1800(0.0) A	F(4,11)= 32.56; P<0.0001
# M 1st series	15.50(25.25)	8.500(16)	0.0(0.75) A,B	4.500(9.25)	0.0(0.0) A,B	F(4,11)=32.02; P<0.0001
# I 1st series	8.000(3.25)	5.500(7)	0.0(0) A	3.0(4.75)	0.0(0.0) A,B	F(4,11)= 31.63; P<0.0001
Latency 1st E (s)	767.5(1341.2)	1642(1228.7)	1800(0) A,B	1800(849.5)	1800(0.0) A,B,D	F(4,11)=22.94; P=0.0001
PEI	411.0(65)	446.0(67)	---------------	373.0(263)	--------------	F(3,11)= 0.6163; P=0.7348
CE_1	37.50(44.25)	19.50(42.75)	0.0(0) A	27.00(35.75)	0.0(0.0) A,D	F(4,11)= 24.31; P<0.0001

M= Mount; I= Intromission; E= Ejaculation; PEL= post-ejaculatory interval; #= number; CE= copulatory efficiency = [# intromissions / (# intromissions + # mounts)] *100

A= Significantly (P<0.05) different from 0 mg/kg. B= Significantly (P<0.05) different from saline + tramadol (20 mg/kg). C= Significantly (P<0.05) different from tramadol (20 mg/kg) + WAY100,635 (0.3 mg/kg). D= Significantly (P<0.05) different from tramadol (20 mg/kg) + naloxone (20 mg/kg).
