# Supplementary material for: Tramadol’s Inhibitory Effects on Sexual Behavior: Pharmacological Studies in Serotonin Transporter Knockout Rats
Source: Front Pharmacol. 2018 Jun 27;9:676. doi: 10.3389/fphar.2018.00676 (PMC6030355; doi:10.3389/fphar.2018.00676)
Supplement: Supplementary file 11 [file Table_11.PDF]

Suppl. table 11: Effects of Tramadol on Sexual Behavior of male SERT$^{+/-}$ Wistar rats.

N=12/group

Dose of tramadol, mg/kg	0 mg/kg A	5 mg/kg B	10 mg/kg C	20 mg/kg D	40 mg/kg E	50 mg/kg	ANOVA significance
Parameters measured	Mean ± SEM	Mean ± SEM	Mean ± SEM	Mean ± SEM	Mean ± SEM	Mean ± SEM	
# E	2.917±0.2876	2.667±0.3553	2.500±0.3794	1.583±0.3128 A	0.7500±0.3509 A,B,C	0.7500±0.4106 A,B,C	F(5,11)= 10.91; P<0.0001
Latency 1st M (s)	84.49±43.88	18.34±3.471	76.94±57.22	364.7±163.9	1230±227.8 A,B,C,D	1353±233.4 A,B,C,D	F(5,11)= 17.07; P<0.0001
Latency 1st I (s)	95.93±46.81	144.7±87.86	223.8±118.6	688.8±197.9	1249±226.2 A,B,C	1360±229.8 A,B,C	F(5,11)= 12.80; P<0.0001
# M 1st series	7.917±2.258	13.00±4.998	9.083±3.716	6.417±1.708	1.917±0.7926	0.5833± 0.5833 B	F(5,11)= 3.040; P< 0.0171
# I 1st series	5.667±0.6317	7.500±0.7538	6.667±0.8196	5.333±1.233	1.917±0.8480 A,B,C,D	1.167±0.6376 A,B,C,D	F(5,11)= 11.07; P<0.0001
Latency 1st E (s)	414.9±122.8	559.2±148.1	542.4±175.5	966.8±189.0	1396±182.6 A,B,C	1421±201.5 A,B,C	F(5,11)= 8.645; P<0.0001
CE_1	54.75±8.592	50.33±7.345	54.50±6.343	46.00±8.456	20.83±8.494 A,B	20.50±11.37 A,B	F(5,11)= 5.074;P= 0.0007

M= Mount; I= Intromission; E= Ejaculation; PEL= post-ejaculatory interval; #= number; CE= copulatory efficiency = [# intromissions / (# intromissions + # mounts)]*100. A= Significantly (P<0.05) different from 0 mg/kg. B= Significantly (P<0.05) different from 5 mg/kg. C= Significantly (P<0.05) different from 10 mg/kg. D= Significantly (P<0.05) different from 20 mg/kg. E= Significantly (P<0.05) different from 40 mg/kg.
