# Supplementary material for: Tramadol’s Inhibitory Effects on Sexual Behavior: Pharmacological Studies in Serotonin Transporter Knockout Rats
Source: Front Pharmacol. 2018 Jun 27;9:676. doi: 10.3389/fphar.2018.00676 (PMC6030355; doi:10.3389/fphar.2018.00676)
Supplement: Supplementary file 12 [file Table_12.PDF]

Suppl. table 12: Effects of WAY100,635 on Sexual Behavior of male SERT$^{+/-}$ Wistar rats.

N=12/group

Dose of WAY10065, mg/kg	0 mg/kg<br>A	0.1mg/kg<br>B	0.3 mg/kg<br>C	2. mg/kg	ANOVA repeated measures significance
Parameters measured	Mean ± SEM	Mean ± SEM	Mean ± SEM	Mean ± SEM	
# E	2.500±0.3589	2.333±0.3553	1.500±0.3989	1.917±0.4345	$F(3,11) = 1.606$; $P=0.2067$
Latency 1st M (s)	49.17±27.77	49.04±14.21	428.0±200.5	463.9±232.6	$F(3,11) = 2.988$; $P=0.0451$
Latency 1st I (s)	155.9±85.69	91.83±36.01	462.4±201.2	495.1±228.2	$F(3,11) = 2.668$; $P=0.0638$
# M 1st series	10.25±2.437	12.42±2.811	14.08±4.217	9.417±3.673	$F(3,11) = 0.5101$; $P=0.6781$
# I 1st series	6.667±0.8288	7.000±0.4924	5.333±1.137	5.667±1.068	$F(3,11) = 0.9039$; $P=0.4497$
Latency 1st E (s)	299.9±49.76	459.2±128.1	898.2±206.3	787.4±217.2	$F(3,11) = 3.732$; $P=0.0205$
PEI	362.5±23.53	433.1±49.98	406.8±42.69	369.0±27.91	$F(3,11) = 0.8268$; $P=0.4889$
CE_1	41.08±5.178	43.25±4.823	31.50±6.861	35.83±6.107	$F(3,11) = 1.152$;$P=0.3428$

M= Mount; I= Intromission; E= Ejaculation; PEL= post-ejaculatory interval; #= number; CE= copulatory efficiency = [# intromissions / (# intromissions + # mounts)]*100. A= Significantly (P<0.05) different from 0 mg/kg. B= Significantly (P<0.05) different from 0.1 mg/kg. C= Significantly (P<0.05) different from 0.3mg/kg.
