# Supplementary material for: Tramadol’s Inhibitory Effects on Sexual Behavior: Pharmacological Studies in Serotonin Transporter Knockout Rats
Source: Front Pharmacol. 2018 Jun 27;9:676. doi: 10.3389/fphar.2018.00676 (PMC6030355; doi:10.3389/fphar.2018.00676)
Supplement: Supplementary file 13 [file Table_13.PDF]

Suppl. table 13: Effects of Naloxone on Sexual Behavior of male SERT +/- Wistar rats.

N=12/group

| Dose of Naloxone, mg/kg | 0 mg/kg A | 5 mg/kg B | 10 mg/kg C | 20 mg/kg | ANOVA repeated measures significance |
|---|---|---|---|---|---|
| Parameters measured | Mean ± SEM | Mean ± SEM | Mean ± SEM | Mean ± SEM | |
| *# E* | 2.667±0.3761 | 2.333±0.4495 | 2.667±0.3098 | 2.833±0.3658 | F(3,11)= 0.4155; P=0.7430 |
| *Latency 1st M (s)* | 14.92±2.527 | 88.83±75.10 | 28.17±8.423 | 165.7±148.6 | F(3,11)= 0.6710; P=0.5759 |
| *Latency 1st I (s)* | 51.58±20.90 | 116.8±90.68 | 32.83±8.976 | 187.8±147.0 | F(3,11)= 0.6857; P=0.5672 |
| *# M 1st series* | 14.33±3.787 | 7.333±1.170 | 10.17±4.199 | 6.250±1.393 | F(3,11)= 1.540; P=0.2226 |
| *# I 1st series* | 6.500±0.7437 | 5.667±0.6435 | 6.667±0.8558 | 5.000±0.9045 | F(3,11)= 1.167; P=0.3370 |
| *Latency 1st E (s)* | 538.3±176.3 | 644.8±204.6 | 427.3±115.6 | 412.1±150.9 | F(3,11)= 0.5006; P=0.6844 |
| *PEI* | 337.0±18.73 | 417.1±37.51 | 390.6±21.57 | 376.8±17.90 | F(3,11)= 1.785; P=0.1665 |
| *$CE_1$* | 44.25±6.851 | 46.08±4.733 | 50.33±4.814 | 43.50±5.800 | F(3,11)= 0.3490; P=0.7901 |

M=Mount; I= Intromission; E=Ejaculation; PEL=post-ejaculatory interval; # =number; CE= copulatory efficiency = [# intromissions / (# intromissions + # mounts)]*100. A= Significantly (P<0.05) different from 0 mg/kg. B= Significantly (P<0.05) different from 5 mg/kg. C= Significantly (P<0.05) different from 10 mg/kg.
