# Supplementary material for: Tramadol’s Inhibitory Effects on Sexual Behavior: Pharmacological Studies in Serotonin Transporter Knockout Rats
Source: Front Pharmacol. 2018 Jun 27;9:676. doi: 10.3389/fphar.2018.00676 (PMC6030355; doi:10.3389/fphar.2018.00676)
Supplement: Supplementary file 14 [file Table_14.PDF]

Suppl. Table 14. WAY100,635 + naloxone effects of tramadol in male SERT$^{+/-}$ rats.

Dose of drug, mg/kg IP	0 mg/kg (saline + saline) A	20 mg/kg Tramadol + Saline B	20 mg/kg Tramadol + WAY 100635 C	20 mg/kg Tramadol + Naloxone D	20 mg/kg Tramadol + WAY 100635 + Naloxone	Friedman test significance
Parameters	Median (IQR)	Median (IQR)	Median (IQR)	Median (IQR)	Median (IQR)	
# E	2.500(1.443)	0.0(1.379)	0.0(1.231) A	0.0(0.9962) A	0.0(0.0) A	F(4,11)= 21.17 ; P<0.0003
Latency 1st M (s)	21.00(21.05)	474.0(805.0)	1714(772.8) A	139.0(840.6)	1800(0.0) A	F(4,11)= 31.17 ; P<0.0001
Latency 1st I (s)	80.50(501.6)	956.0(791.5)	1800(779.2)	805.0 (827.0)	1800(0.0) A	F(4,11)= 20.52 ; P= 0.0004
# M 1st series	14.00(7.692)	2.500(4.048)	0.5000(5.485)	0.5000(4.239)	0.0(0.0) A	F(4,11)= 25.62 ; P<0.0001
# I 1st series	5.000(2.552)	3.000(2.644)	0.0(2.927)	2.500(1.782)	0.0(0.0) A	F(4,11)= 31.63; P<0.0001
Latency 1st E (s)	421.0(639.8)	1800(785.7)	1800(699.8)	1800(631.9)	1800(0.0) A	F(4,11)= 21.19 ; P=0.0003
PEI	373.0(73.24)	467.0(45.86)	465.0(120.3)	468.5(48.79)	--------------	F(3,11)= 4.492 ; P= 0.2130
CE_1	35.00(16.39)	27.50(26.01)	0.0(27.04)	27.00(36.76)	0.0(0.0) A	F(4,11)= 15.28; P<0.0042

M= Mount; I= Intromission; E= Ejaculation; PEL= post-ejaculatory interval; #= number; CE= copulatory efficiency = [# intromissions / (# intromissions + # mounts)] *100. A= Significantly (P<0.05) different from 0 mg/kg. B= Significantly (P<0.05) different from saline + tramadol (20 mg/kg). C= Significantly (P<0.05) different from tramadol (20 mg/kg) + WAY100,635 (0.3 mg/kg). D= Significantly (P<0.05) different from tramadol (20 mg/kg) + naloxone (20 mg/kg).
