# Supplementary material for: Tramadol’s Inhibitory Effects on Sexual Behavior: Pharmacological Studies in Serotonin Transporter Knockout Rats
Source: Front Pharmacol. 2018 Jun 27;9:676. doi: 10.3389/fphar.2018.00676 (PMC6030355; doi:10.3389/fphar.2018.00676)
Supplement: Supplementary file 15 [file Table_15.PDF]

1.Supplementary figures titles and legends for SERT $^{+/-}$ animals.

Supplementary Figure 1. Effects of tramadol in male SERT$^{+/-}$ rats. N=12 animals/group

Supp. Figure 1. Sexual behavior of SERT$^{+/-}$ male rats (N=12/ group) treated with 0, 10, 20, 40 or 50 mg/kg tramadol. Data are given as mean ± SEM. The number and latency of ejaculations per 30 min (A, D) and latency of Mounts (B, E), number and latency of Intromissions (C, F) of the first Ejaculation Series are given. Detailed statistical analyses (ANOVA repeated measures) are shown in Suppl. Tables 9. a: significant difference (P<0.05) compared to saline group.

Supplementary Figure 2. Effect of WAY 100635 in male SERT$^{+/-}$ rats. N=12 animals/group

Supp. Figure 2. Sexual behavior of SERT$^{+/-}$ male rats (N=12/group) treated with saline, WAY,100635 0.01 mg/kg, WAY100,635 0.03 mg/kg or WAY100,635 3 mg/kg. Data are given as mean ±SEM. The number and latency of ejaculations per 30 min (A, D), number and latency of Mounts (B, E), number and latency of Intromissions (D, F). Detailed statistical analyses (ANOVA repeated measures) are shown in Suppl. Table 10. a: significant difference (P<0.05) compared to saline group.

Supplementary Figure 3. Effect of naloxone in male SERT$^{+/-}$ rats. N=12 animals/group

Supp. Figure 3. Sexual behavior of SERT$^{+/-}$ male rats (N=12/group) treated with saline, Naloxone 5 mg/kg, Naloxone 10 mg/kg or Naloxone 20 mg/kg. Data are given as mean ± SEM. The number and latency of ejaculations per 30 min (A, D), number and latency of Mounts (B, E), number and latency of Intromissions (C, F). Detailed statistical analyses (ANOVA repeated measures) are shown in Suppl. Table 11. a: significant difference (P<0.05) compared to saline group.

Supplementary Figure 4. WAY100,635 + naloxone + tramadol effects in male SERT$^{+/-}$ rats. N=12 animals/group

Supp. Figure 4. Sexual behavior of SERT$^{+/-}$ male rats (N=12/group) treated with saline + saline (S+S), tramadol (20 mg/kg) + saline (T+S), tramadol (20 mg/kg) + WAY100,635 (0.3 mg/kg; T+W), tramadol (20 mg/kg) + Naloxone (20 mg/kg; T+N) and tramadol (20 mg/kg) + WAY100,635 (0.3 mg/kg) + Naloxone (20 mg/kg; T+W+N). The number and latency of ejaculations per 30 min (A, D), number and latency of Mounts (B, E), number and latency of Intromissions (C, F), and Intromission Rate (G). Detailed statistical analyses (ANOVA repeated measures) are shown in Suppl. Table 12. a: significant difference (P<0.05) compared to saline + saline group.
