# Supplementary figures and images for: Tramadol’s Inhibitory Effects on Sexual Behavior: Pharmacological Studies in Serotonin Transporter Knockout Rats
Source: Front Pharmacol. 2018 Jun 27;9:676. doi: 10.3389/fphar.2018.00676 (PMC6030355; doi:10.3389/fphar.2018.00676)

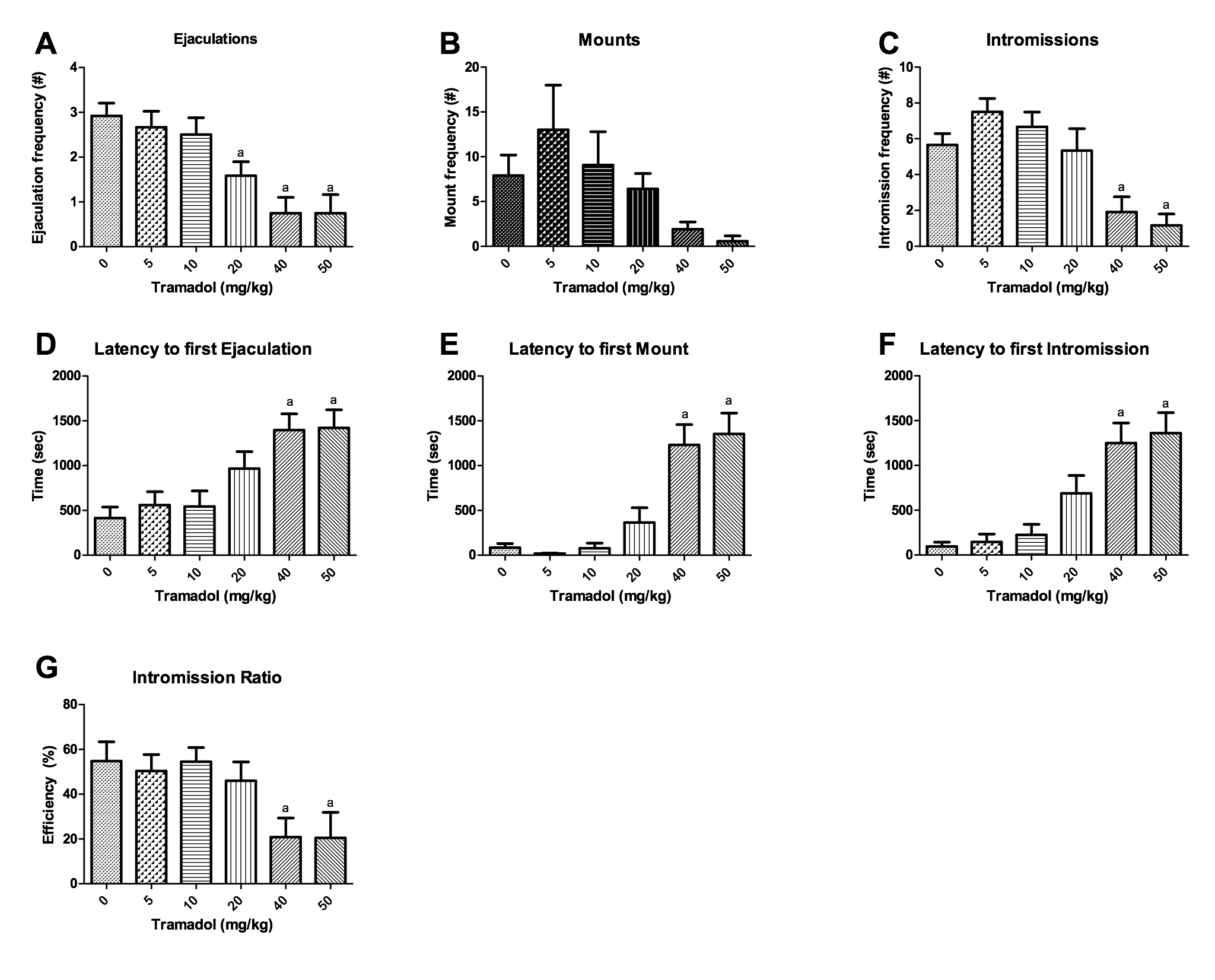

Supplement: Supplementary file 16 [file Image_1.TIF]

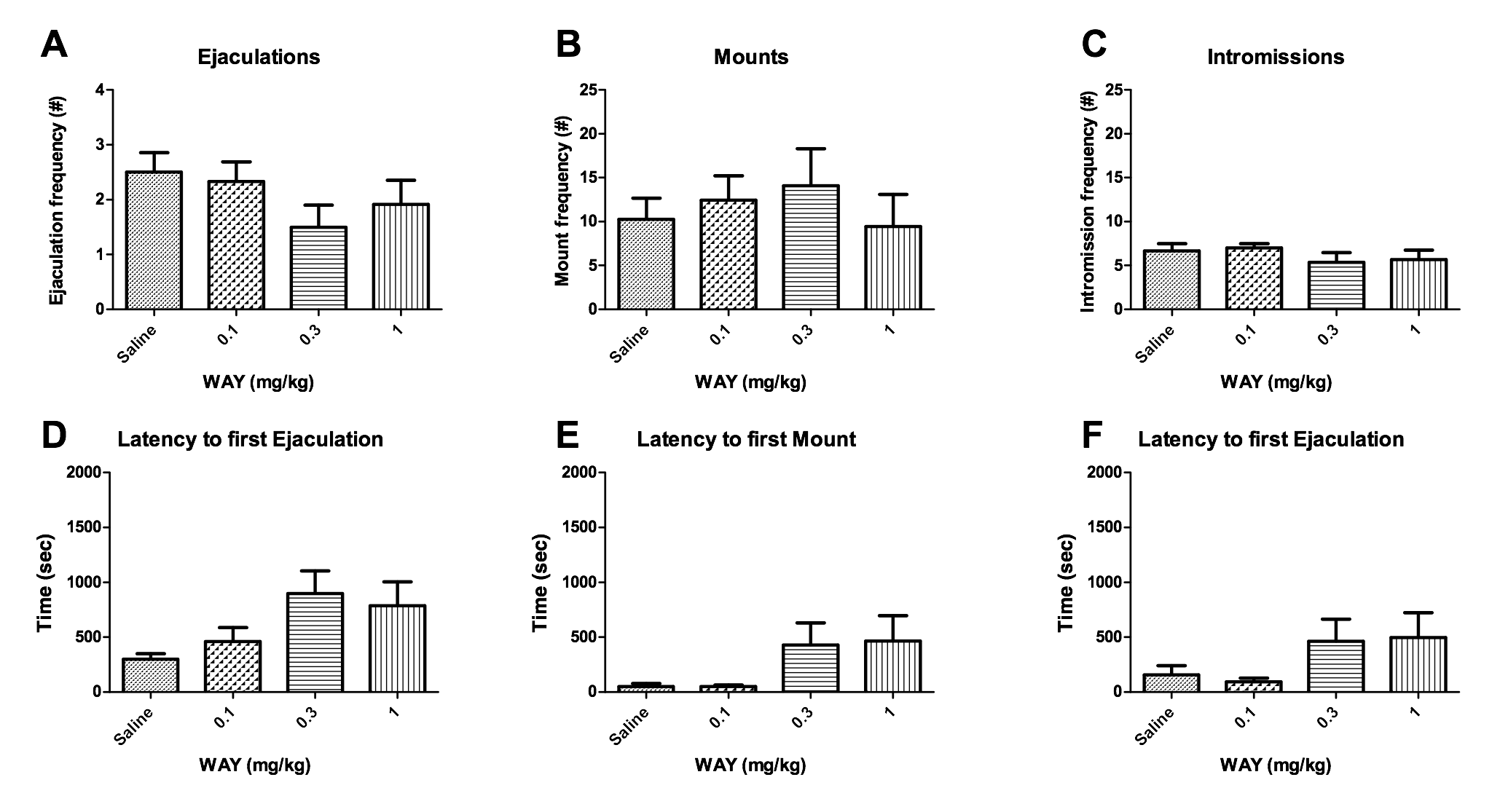

Supplement: Supplementary file 17 [file Image_2.TIF]

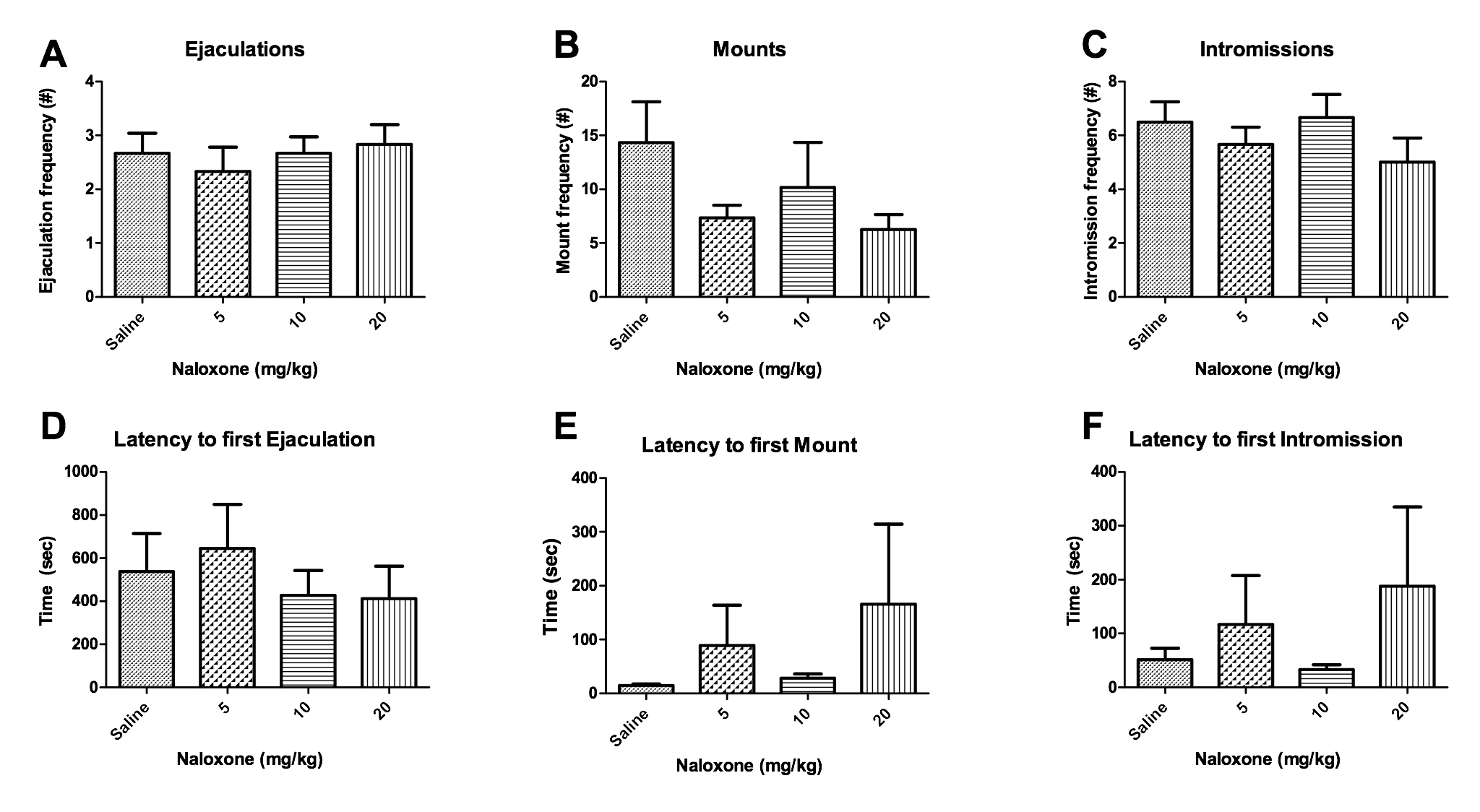

Supplement: Supplementary file 18 [file Image_3.TIF]

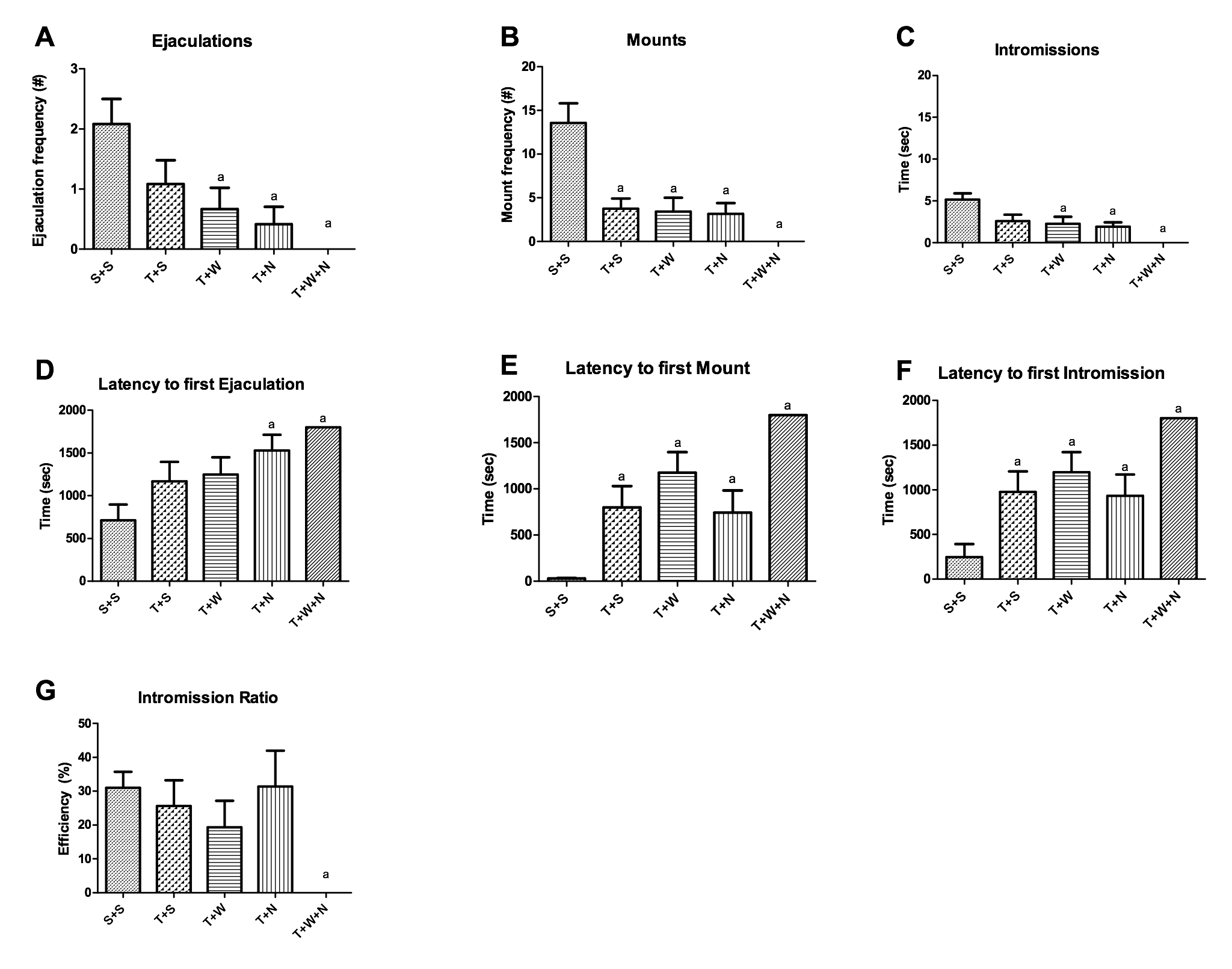

Supplement: Supplementary file 19 [file Image_4.TIF]
